# An assessment of a conservation strategy to increase garden connectivity for hedgehogs that requires cooperation between immediate neighbours: A barrier too far?

**DOI:** 10.1371/journal.pone.0259537

**Published:** 2021-11-05

**Authors:** Abigail Gazzard, Anne Boushall, Emma Brand, Philip J. Baker

**Affiliations:** School of Biological Sciences, University of Reading, Whiteknights, Reading, Berkshire, United Kingdom; University of Oklahoma Norman Campus: The University of Oklahoma, UNITED STATES

## Abstract

Urban areas are associated with high levels of habitat fragmentation. For some terrestrial species with limited climbing abilities, property boundaries can pose a significant problem by limiting access to residential gardens. The West European hedgehog (*Erinaceus europaeus*) has declined markedly in the UK but is commonly found in areas of human habitation, including residential gardens. ‘Hedgehog Street’ is a public engagement campaign aimed at recruiting volunteers (‘Hedgehog Champions’) to create access points (‘hedgehog highways’) across garden boundaries to improve habitat connectivity. In this study, we used a series of questionnaire surveys to explore motivations for and obstacles to the creation of highways. Householders were more likely to have created a highway if they were already aware of the Hedgehog Street campaign, if their garden contained a high number of wildlife-friendly features and if they considered watching wildlife to be important. Hedgehog Champions created, on average, 1.69 highways each with 52.0% creating none; this would equate to an estimated >120,000 across all registered Champions. In comparison, 6.1–29.8% of non-Champions stated that they had made a highway. However, most highways had been created in boundaries that could already be traversed via naturally occurring holes: only 11.4% of garden boundaries could be traversed, and 3.2% of gardens accessed, just via a hedgehog highway. In addition, only 5.0% of gardens were considered totally inaccessible to hedgehogs. The most common reasons cited for not having made a highway were that householders’ gardens were already accessible to hedgehogs followed by concerns relating to boundary ownership and / or communicating with neighbours. Future studies need to identify strategies for overcoming these obstacles to maximize citizen engagement, particularly with those householders who are not innately “wildlife-friendly”, and to quantify the degree to which networks of highways affect patterns of individual movement and, ultimately, populations.

## Introduction

Urbanisation is a major form of anthropogenic land-use change and is typically associated with a decline in biological diversity [1–4]. Such declines are effects of the destruction, degradation and fragmentation of natural / semi-natural habitats but also the presence of a range of characteristics associated with urban areas that many species cannot tolerate [4, 5]. Consequently, ecological communities in urban areas are often dominated by generalist species [6, 7], with some occurring at higher densities in towns and cities than in natural habitats [8–12]. Urban areas can, nonetheless, support species-rich assemblages [13–16], including species of conservation concern [17–19]. As such, urban areas could function as a conservation tool for wildlife if managed sympathetically [16, 20–22].

The physical structure of urban areas varies markedly between countries [23]. In the UK, they contain a wide range of natural and semi-natural green- and blue-spaces but are dominated by private residential gardens [24]. Individually, gardens tend to be small but collectively cover a substantial area. For example, Davies *et al*. [25] estimated a mean garden size of 190m^2^ which, multiplied across the estimated 22.7 million UK households with access to a garden, equates to a combined area of >4,000 km^2^. Residential gardens therefore offer potentially substantive conservation benefits, yet present considerable challenges such as the possible need to engage large numbers of householders for these benefits to be realised [26, 27].

Many UK householders are interested in wildlife as demonstrated by the millions of ponds and nest boxes installed in residential gardens, and the fact that approximately 51% of residents supply food for birds at least some of the time [25]. However, wildlife gardening activities are often directed at species which are very mobile or not of conservation concern. For example, bird feeders are often utilised by species that are common and widespread or non-native [28, 29]. One corollary of high mobility is that neighbouring householders do not necessarily need to coordinate their wildlife-gardening efforts as fauna can fly between gardens or climb over / dig under garden boundaries. For less agile species, however, coordination between neighbours becomes more critical.

The West European hedgehog (*Erinaceus europaeus;* hereafter ‘hedgehog’) is a small (<1.5kg), cursorial, nocturnal mammal which has declined markedly in Britain and Europe in recent decades [30–33]. In rural landscapes, primary threats include habitat loss, fragmentation and degradation [34–36] and an increase in the number of badgers (*Meles meles*) [37], an intraguild predator [38]. As a result, hedgehogs are now increasingly found within or near human settlements [11, 39–41], with residential gardens (especially rear gardens) a favoured habitat [41, 42]. However, urban-dwelling hedgehogs face a range of challenges including accidental exposure to pesticides [43], human disturbance [44], injury by domestic animals [33], and barriers to movement including roads [45] and garden fences [31]. The latter is considered of increasing importance because of perceived changes in the numbers of rear gardens fully or partially enclosed by wooden fences, particularly those with gravel boards (horizontal wooden or concrete boards at ground level designed to protect fence panels from ground-level moisture; these have the effect of reducing the number of holes in fences caused by the natural deterioration of the fence material). To this end, two UK charities, the People’s Trust for Endangered Species (PTES) and the British Hedgehog Preservation Society (BHPS), launched the citizen engagement program ‘Hedgehog Street’ in 2011 to aid the conservation of urban hedgehog populations.

### Hedgehog Street, hedgehog highways and Hedgehog Champions

Hedgehog Street (HS) is administered via a website (www.hedgehogstreet.org) that summarises information on hedgehog ecology and behaviour, trends in hedgehog numbers and how people can make their gardens more hedgehog-friendly. The website also acts as a forum for people to share information, observations and photographs. Individuals are encouraged to engage with the program by signing up to become a ‘Hedgehog Champion’ (hereafter ‘Champion’).

One major focus of HS is to persuade members of the public (Champions and non-Champions) to create holes (130*130mm) through or under their garden boundaries (‘hedgehog highways’; hereafter ‘highways’) to increase connectivity between gardens. These could potentially help hedgehogs in three ways: (i) enabling entry to previously inaccessible gardens, thereby increasing the carrying capacity of the environment; (ii) reducing travel distances between gardens, thereby reducing the energetic burden of foraging; and / or (iii) reducing the number of road crossings between blocks of houses, thereby reducing the mortality risk from traffic. These putative benefits are, however, predicated on several key assumptions e.g. that currently inaccessible gardens contain resources that hedgehogs require, and that highways do not simply allow animals to traverse boundaries where crossing points already exist. Although comprehensive evidence on the effectiveness of improving inter-garden connectivity is lacking, local field studies have demonstrated that hedgehog detection and occupancy rates are influenced to varying degrees by garden accessibility [46, 47].

Given that urban hedgehog populations need up to 90ha of suitable habitat for numbers to be sustainable [31], and that individuals may visit up to 20 gardens nightly [33], relatively large numbers of highways would need to be constructed in a single neighbourhood to generate an effect of significant magnitude to positively influence hedgehog density, survival rates and / or reproductive output. Accordingly, Champions are given access to additional support materials to help them enlist other householders with the goal of creating a high-density network of highways in their neighbourhood. Champions and non-Champions are also asked to upload geo-referenced sightings of hedgehogs (dead or alive) and the position of any highways that they have created to an interactive map (The Big Hedgehog Map: www.bighedgehogmap.org). At the time of writing (October 2021), >100,000 people have signed up as Champions, and >100,000 and >18,000 sightings of live and dead hedgehogs have been reported, respectively, as well as the creation of >15,000 highways.

Despite the apparently high levels of engagement with this campaign, and the public’s generally positive attitude towards hedgehogs [48–52], UK urban hedgehog populations are still declining [53]. The reasons for this are likely to be multi-faceted, but could be partly associated with the ability of citizens to engage with hedgehog conservation strategies, even if they are willing. For example, HS requires immediate neighbours to create a highway through or under a shared garden boundary, and this is subtly different from most other wildlife-friendly gardening practices since: it requires communication and agreement between neighbouring householders to avoid disputes (householders typically own the rights to just one of the boundaries running down the side of their property); it involves the alteration of a boundary structure which may have been erected to maintain privacy or to keep pets within the owner’s garden; and it might be considered aesthetically unpleasing. In addition, residents may not own the property they are living in; approximately 34% of UK households are privately or socially rented houses [54] and tenants may not be permitted to modify any boundaries.

It is also reasonable to expect that not all householders are concerned about the plight of hedgehogs, whereas others may have their own perceptual biases about the need to create highways. For example, residents that have already seen hedgehogs in their garden may consider that creating further access points is unnecessary, whilst not appreciating that these could offer additional advantages in terms of movement through the wider landscape. Furthermore, householders that never see hedgehogs in their garden / neighbourhood may conclude that hedgehogs are simply not present, even though this may not be the case. Consequently, the HS campaign could be associated with a number of significant challenges and, as with other conservation campaigns, should ideally be managed adaptively [55–58]. This means that progress needs to be assessed periodically with a view to amending, or even abandoning, strategies if deficiencies are evident [56, 59, 60]. Therefore, in this study we used a series of questionnaire surveys to: (1) quantify the proportions of Champions and non-Champions who have created a highway; (2) identify the factors associated with the creation of highways; (3) examine the relative importance of reasons given for not having created a highway; (4) estimate the potential effect of the creation of these highways on hedgehog movement patterns; and (5) outline recommendations for the future growth of this campaign.

## Materials and methods

Data were collected through a series of online questionnaires in September-October 2018, October 2019 and December 2019-April 2020; these are referred to as the 2018, 2019 and 2020 surveys, respectively (S1–S3 Files). The first two surveys were conducted in collaboration with University of Reading students as part of their undergraduate studies; online links to each questionnaire were advertised via postings on relevant social media groups (e.g. those related to gardening and wildlife, as well as local community groups) and released to family members of all students within the School of Biological Sciences with instructions for them to disseminate it to further friends and family.

The 2020 survey was conducted in collaboration with the PTES and BHPS and released to all Hedgehog Champions registered to receive email communications at that time (N = 43,650), as well as social media followers of PTES and BHPS. Since it was possible for non-Champions to take part in this survey via the links provided on social media, respondents were asked to clarify whether they were registered as Champions or not. Given the slight differences between surveys, we have selected and / or merged responses from individual surveys where necessary.

Surveys were granted approval by the ethical review panel of the School of Biological Sciences at the University of Reading. At the start of each survey, respondents were informed of the goals of the survey, how the data would be stored and used, that the data would not be shared with any third party and that the data would be anonymous (i.e. it would not be possible to identify any individual from the information supplied). Respondents provided written informed consent and were also asked to confirm that they were aged 18 or over before being granted access to the questionnaire itself.

### Proportion of respondents creating hedgehog highways

Survey data were used to derive three estimates of the proportion of Champions (P_C_) and non-Champions (P_N_) who had made a highway. As respondents in the 2020 survey were asked whether they had registered as a Hedgehog Champion, estimates for P_C_ and P_N_ were derived from those respondents that stated that they were and were not registered Champions, respectively.

In the 2018 and 2019 surveys, respondents were asked whether they had heard of the HS campaign, but not whether they had registered as a Champion. Consequently, each data set could have consisted of a combination of Champions and non-Champions. Therefore, data from respondents that had not heard of the HS campaign were used to estimate P_N_ (by inference these respondents could not have signed up to become a Champion), whereas data from respondents that had heard of HS were used to estimate P_C_ (this assumes that these respondents may have signed up to become Champions). Estimates for both parameters were derived from the 2018 and 2019 surveys separately. Differences in P_C_ and P_N_ between surveys were compared using chi-squared tests; *post hoc* groups were identified using the procedure outlined by Siegel and Castellan [61].

To investigate possible biases in the households surveyed, we used a series of chi-squared tests to compare the proportion of respondents that fed birds, had a bird box and / or pond in their garden with the corresponding proportions cited by Davies *et al*. [25] for the UK (51%, 21% and 16%, respectively). These analyses compared: (i) all individuals in each of the three surveys; (ii) those respondents who had / had not made a hedgehog highway; and (iii) those respondents who had / had not heard of the Hedgehog Street campaign. A Bonferroni correction was applied to adjust for multiple testing.

In addition, all respondents were asked whether they fed hedgehogs or had a hedgehog house in their garden. Champions were further asked whether they had created their highway before or after they knew hedgehogs were present in their garden and whether they thought hedgehog activity in their garden had increased after having created a highway.

### Hedgehog accessibility into neighbouring gardens

Patterns of accessibility into back gardens and across individual boundaries between neighbouring gardens were quantified using data from 2019 and 2020. In both surveys, householders were asked to state: (1) the number of neighbouring gardens bordering their own back garden; (2) the number of these gardens that were accessible to hedgehogs via (i) a natural hole only (e.g. a hole that had been dug under the fence by an animal or a hole in the fence caused by natural deterioration), (ii) a highway only, and (iii) via a combination of both natural holes and highways; and (3) whether their back garden could be accessed by a hedgehog from their front garden. These data were used to identify how many gardens were totally inaccessible to hedgehogs, how many gardens were accessible via highways only, and how many boundaries could be traversed via highways only.

Hedgehog Champions who had made a highway were also asked to provide information on the number of additional householders that they had successfully recruited into making highways in their immediate neighbourhood (defined as a contiguous set of houses on the householder’s street where the back gardens were linked) and further afield.

### Factors affecting the decision to have made a hedgehog highway

The questionnaires requested information on whether householders had created ≥1 highways in their garden (HIGHWAY) as well as variables considered to potentially influence this decision: the respondent’s physical location (geographical REGION and HOUSESETTING); the number of people living at the house (RESIDENTS); the length of time that they had been living at the house (YEARSRESIDED); the type of house they lived in (HOUSETYPE); the respondent’s level of employment (EMPLOYMENT); whether they had a front garden, back garden, communal garden or a combination of these (GARDENTYPE); whether their garden contained wild flowers (FLOWERS), water that could be accessed by wildlife (excluding a pond: WATER) and / or a flowering LAWN, wild PATCH, hedgerow (HEDGE), LOGPILE, POND, BIRDBOX, BATBOX, HEDGEHOGHOUSE, insect HOTEL and COMPOST heap; whether they had sighted badgers (BADGER), foxes (*Vulpes vulpes*; FOX), rodents (RODENT) and / or hedgehogs (HEDGEHOG) in their garden in the previous 12 months; whether they left food out for hedgehogs (FEEDHEDGEHOG); whether they had heard of Hedgehog Street prior to the survey (HEDGEHOGSTREET); and whether they belonged to any wildlife or environmental groups (ENVIGROUPS). Because of the small number of cases in some categories, the variables BADGER and FOX were merged to indicate whether the respondent had sighted badgers or foxes in their garden in the previous 12 months (BADGERFOX), and the 12 variables FLOWERS-COMPOST outlined above were tallied to create a binary variable indicating low (≤6 features) or high (>6) numbers of wildlife-friendly GARDENFEATURES in the respondent’s garden.

To consider differences in how people may value wildlife in their gardens versus using their garden for other activities, respondents were asked to rank how important they considered each of the following ten activities: watching birds, watching other wildlife, gardening, growing their own food, socialising, relaxing, use by pets, use by children, for drying laundry and for storage. All variables were measured using a four-point Likert scale: less important, somewhat important, important and very important, with data coded as 1–4 respectively. Values were then averaged across subsets of these ten activities to create three variables: WATCHWILDLIFE (mean of watching birds and other wildlife); GARDENING (mean of gardening and growing own food); and RECREATION (mean of socialising, relaxing, use by pets and children, drying laundry and storage). Scores >2 and ≤2 indicated that the activity was or was not important to the respondent, respectively. All variables are summarised in Table 1.

**Table 1:** Summary of variables requested in the 2018, 2019 and 2020 surveys that were used to investigate the factors affecting a householder’s decision to create a hedgehog highway.

Name	Description	Levels
HIGHWAY	Dependent variable; a binary measure of whether the respondent had made a hedgehog highway or not	(0) No
		(1) Yes
RESIDENTS	Number of residents occupying the address at the time of the survey	Continuous
YEARSRESIDED	The length of time that the address had been occupied by the respondent	(1) 0–5 years
		(2) 6–20 years
		(3) >21 years
REGION	The region of the UK where the respondent lived	(1) East
		(2) Southeast
		(3) Southwest
		(4) Northwest
		(5) London
		(6) East Midlands
		(7) Northeast
		(8) Yorkshire and the Humber
		(9) West Midlands
		(10) Wales
		(11) Scotland
		(12) Northern Ireland
SETTING	Type of location where house is situated	(0) In a village or smaller
		(1) In a town or city
HOUSETYPE	Type of house	(1) Detached
		(2) Semi-detached
		(3) Terraced
		(4) Flat
GARDENTYPE	Extent / type of gardens associated with property	(1) One private front garden OR one private back garden OR communal garden
		(2) Both a private front AND back garden
GARDENFEATURES	Extent of wildlife-friendly features present within respondent’s garden, selected from multiple-choice options (flowering lawn; wildflowers; wild patch; hedgerow; log pile; pond; bird box; bat box; hedgehog house; insect hotel; compost heap; water for wildlife)	(0) Six or less features
		(1) Seven or more features
BADGERFOX	Whether the respondent had sighted a badger or fox in their garden in 12 months prior to the survey [NB badger and fox sightings were merged due to the low number of positive sightings]	(0) Not sighted in last 12 months
		(1) Sighted in last 12 months
HEDGEHOG	Whether the respondent had sighted a hedgehog in their garden in 12 months prior to the survey	(0) Not sighted in last 12 months
		(1) Sighted in last 12 months
RODENT	Whether the respondent had sighted a rodent in their garden in 12 months prior to the survey	(0) Not sighted in last 12 months
		(1) Sighted in last 12 months
HEDGEHOGSTREET	Whether the respondent had heard of Hedgehog Street prior to the survey	(0) Not aware
		(1) Aware
FEEDHEDGEHOG	Whether the respondent ever leaves food out for hedgehogs in their garden	(0) Does not leave food out
		(1) Leaves food out
ENVIGROUPS	Whether the respondent was a member of any environmental or wildlife groups	(0) Not a member
		(1) Is a member
EMPLOYMENT	Respondent’s level of employment	(1) Part-time
		(2) Full-time
		(3) Unemployed or homemaker
		(4) Student
		(5) Retired
		(6) Prefer not to say / other
WATCHWILDLIFE	A ranking of how important the respondent considered garden wildlife-watching activities to be (averaged from the variables ‘watching birds’ and ‘watching other wildlife’)	(0) Less important or not important
		(1) Important or very important
GARDENING	A ranking of how important the respondent considered gardening to be (averaged from the variables ‘gardening’ and ‘growing food’)	(0) Less important or not important
		(1) Important or very important
RECREATION	A ranking of how important the respondent considered recreational uses of the garden to be (averaged from the variables ‘socialising’, ‘relaxing’, ‘use by pets’, ‘use by children’, ‘laundry’ and ‘storage’)	(0) Less important or not important
		(1) Important or very important

Generalised linear models (GLM) with binomial distributions were used to examine factors affecting people’s decisions to have made a highway in R (version 4.0.3). Although the choice to make a highway may have been a household decision, we included individual-level variables in the analyses because of the impracticalities surrounding questioning all household members. The interaction term HOUSETYPE*HOUSESETTING was included as it was theorised that any effect of house type might be dependent on whether the house was in an urban (town or city) or rural (village or smaller) setting.

Candidate models were constructed through an iterative process of variable selection whereby covariates were added successively and the Akaike’s Information Criterion (AIC) of each model compared. The data were examined for multicollinearity using Generalized Variance Inflation Factors (GVIF) in the form GVIF^(1/(2*Df))^ [62]. Variables that were not significant, but which improved model fit, were retained. Optimal models were selected by comparing AIC values and model fit using Hosmer and Lemeshow and pseudo- R^2^ values [63].

Three of the variables listed in Table 1 (RODENT, HEDGEHOG, FEEDHEDGEHOG) could potentially complicate interpretation of the results of this analysis as they may have influenced a householder’s decision to create a hedgehog highway in the first instance, or they may have changed as a result of the creation of a highway. Therefore, we present two final models: one including these three variables and one where they have been excluded.

### Reasons cited for not having made a hedgehog highway

Respondents in all three surveys who had not made a highway were asked to indicate why they had not done so from a list of 11 possible reasons: I am not interested; I don’t want to damage the boundary structure; I don’t want to speak to my neighbour or carry out works to their boundary structure; it would be unsightly; it might encourage rats; there are no hedgehogs where I live; small pets might escape; I rent my property; I don’t have enough time; my garden is already accessible to hedgehogs; I don’t have the correct tools and / or don’t know how to make a highway. Respondents were able to select multiple reasons and outline any “other” possible underlying reason(s) as well.

## Results

Responses were received from 5986 individuals (2018: N = 506; 2019: N = 402; 2020: N = 5078; S4). Overall, 4759 respondents in the 2020 survey (93.7%) confirmed that they were registered as Champions, giving a response rate for Champions of 6.7% (N = 71,166 Champions registered at the time of surveying in December 2019). Of those, 2285 (P_C_ = 48.0%) had created at least one highway in their own garden. This figure was significantly different to the corresponding proportions of respondents who had made a highway: (i) in both the 2018 (P_N_ = 19.1%, N = 241) and 2019 (P_N_ = 6.1%, N = 230) surveys but stated that they had not heard of the HS campaign (X^2^_2_ = 223.66, P < 0.001; overall P_N_ = 12.7%, N = 471); (ii) who stated that they had heard of HS in the 2018 (P_C_ = 56.2%, N = 265) and 2019 (P_C_ = 20.3%, N = 172) surveys (X^2^_2_ = 59.44, P < 0.001; overall P_C_ = 42.1%, N = 437); and (iii) those who were not Champions in the 2020 survey (P_N_ = 29.8%, N = 319) (X^2^_1_ = 39.92, P < 0.001).

In general, a significantly greater proportion of respondents within each of the three surveys fed birds frequently (except in 2019), had a bird box and / or a pond in their garden compared to the national figures reported by Davies *et al*. [25] (Table 2). This was also the case when the data were partitioned into those respondents who had and who had not made a highway, and those who had heard of HS; however, there was no significant difference with regards feeding birds and having a pond for that subset of respondents who had not heard of HS (Table 2). Many respondents also reported that they fed hedgehogs frequently (2018: 19.4%; 2019: 9.2%; 2020: 66.2%) and / or had a hedgehog box in their garden (2018: 25.1%; 2019: 12.4%; 2020: 68.0%).

**Table 2:** Comparisons of the number of respondents (N = 5986) who (a) fed birds frequently, (b) had a bird box or (c) pond in their garden, relative to the estimates reported for the UK population by Davies *et al*. [25].

Grouping	Garden wildlife factor	Observed	Expected	X^2^_1_	*p*	SIG
**2018**	Fed birds	307 (0.61)	258.06 (0.51)	9.28	0.002	*
**(N = 506)**						
	Bird box	286 (0.57)	106.26 (0.21)	304.03	< 0.001	*
	Pond	145 (0.29)	80.96 (0.16)	50.66	< 0.001	*
**2019**	Fed birds	224 (0.56)	205.02 (0.51)	1.76	0.185	
**(N = 402)**	Bird box	170 (0.42)	84.42 (0.21)	86.76	< 0.001	*
	Pond	93 (0.23)	64.32 (0.16)	12.79	< 0.001	*
**2020**	Fed birds	4336 (0.85)	2589.78 (0.51)	1177.43	< 0.001	*
**(N = 5078)**	Bird box	3611 (0.71)	1066.38 (0.21)	6072.03	< 0.001	*
	Pond	2151 (0.42)	812.48 (0.16)	2151.75	< 0.001	*
**Made highway**	Fed birds	2328 (0.89)	1338.24 (0.51)	732.02	< 0.001	*
**(N = 2624)**	Bird box	2009 (0.76)	551.04 (0.21)	3857.52	< 0.001	*
	Pond	1212 (0.46)	420 (0.16)	1493.49	< 0.001	*
**Not made highway**	Fed birds	2539 (0.76)	1714.62 (0.51)	396.36	< 0.001	*
	Bird box	2058 (0.61)	706.02 (0.21)	2588.95	< 0.001	*
**(N = 3362)**						
	Pond	1177 (0.35)	537.92 (0.16)	759.26	< 0.001	*
**Heard of HS**	Fed birds	4638 (0.84)	2812.65 (0.51)	1184.61	< 0.001	*
**(N = 5515)**	Bird box	3958 (0.72)	1158.15 (0.21)	6768.69	< 0.001	*
	Pond	2299 (0.42)	882.4 (0.16)	2274.20	< 0.001	*
**Not heard of HS**	Fed birds	229 (0.49)	240.21 (0.51)	0.52	0.470	
**(N = 471)**	Bird box	193 (0.41)	98.91 (0.21)	89.50	< 0.001	*
	Pond	90 (0.19)	75.36 (0.16)	2.84	0.092	

Chi-squared test results are provided for all survey years (2018, 2019, 2020), for those respondents who had or had not made a hedgehog highway at the time of surveying, and those who had or had not heard of the Hedgehog Street campaign. Figures in parentheses in the observed and expected columns are the proportion of respondents.

* indicates difference is significant (p < 0.05) after applying Bonferroni correction (0.05 / 21 = 0.002) for multiple testing.

Of the 2285 Champions that had created a highway, 1681 (73.8%) had done so after they knew that hedgehogs were visiting their garden, with 1226 (53.7%) stating that they had subsequently observed an increase in hedgehog activity in their garden.

### Hedgehog accessibility into neighbouring gardens

**Fig 1:**
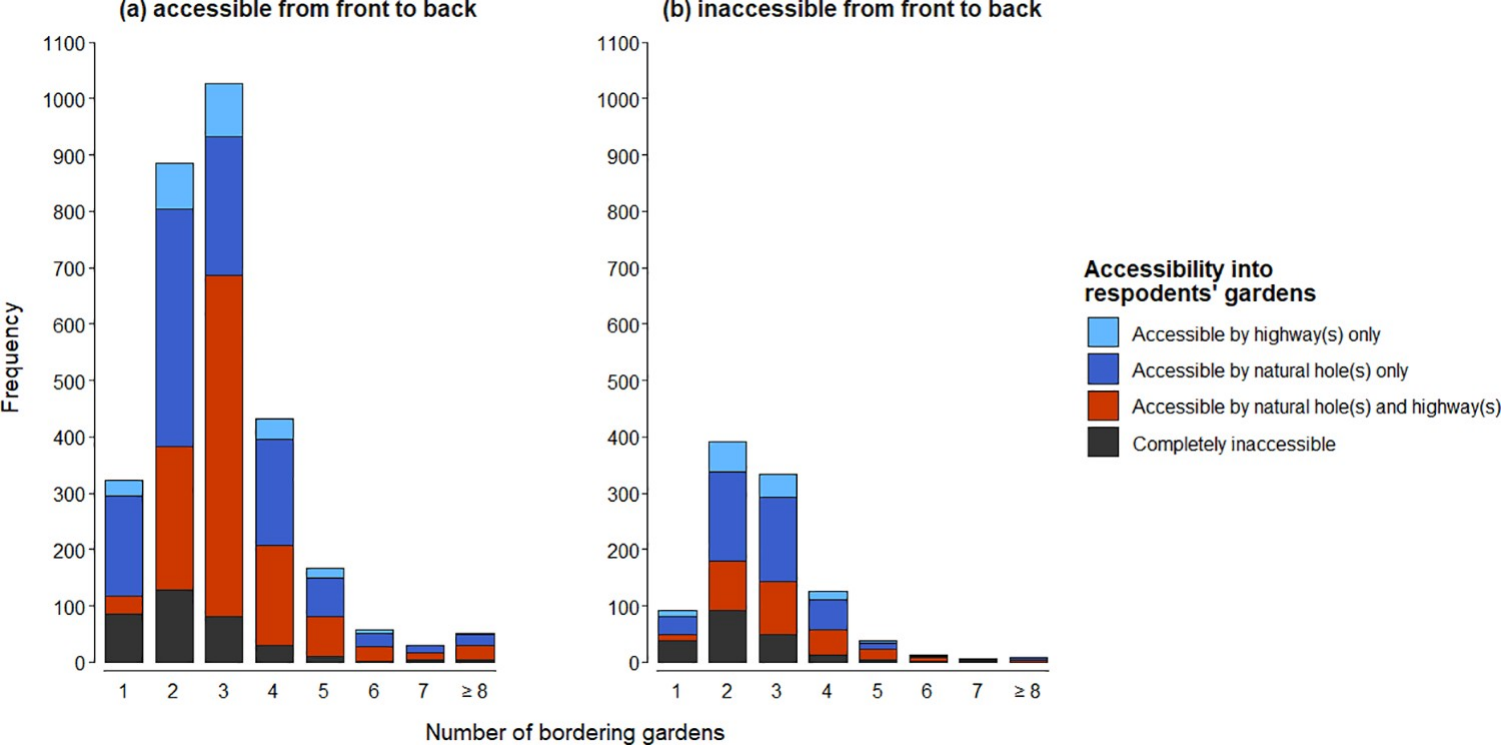
The number of respondents’ back gardens that were accessible to hedgehogs via hedgehog highways and / or naturally occurring holes in relation to the number of bordering gardens. Data are split into those back gardens which were (a) accessible (N = 2969) and (b) not accessible (N = 1009) from the respondent’s own front garden.

Overall, 3978 respondents bordered by ≥1 back garden(s) provided information about accessibility into their own back garden; 118 respondents had no neighbouring back gardens. Of the former, 2969 (74.6%) were (Fig 1A) and 1009 (25.4%) were not (Fig 1B) accessible via the respondent’s front garden. Collectively, 543 respondents (13.7%) indicated that they thought hedgehogs could not access their back garden from neighbouring back gardens. However, 345 of these gardens were accessible from the respondent’s own front garden (Fig 1A). Consequently, only 198 (5.0%) gardens were considered completely inaccessible to hedgehogs (Fig 1B).

The back gardens of 1574 respondents (40.0% of bordered gardens) were accessible from neighbouring back gardens only via natural holes, 1469 (36.9%) were accessible via a combination of natural holes and hedgehog highways, and 392 (9.9%) were accessible only via highways. Of the latter, however, 264 could also be accessed via a front garden (Fig 1A), indicating that highways only granted access to 128 (3.2%) previously inaccessible back gardens.

**Fig 2:**
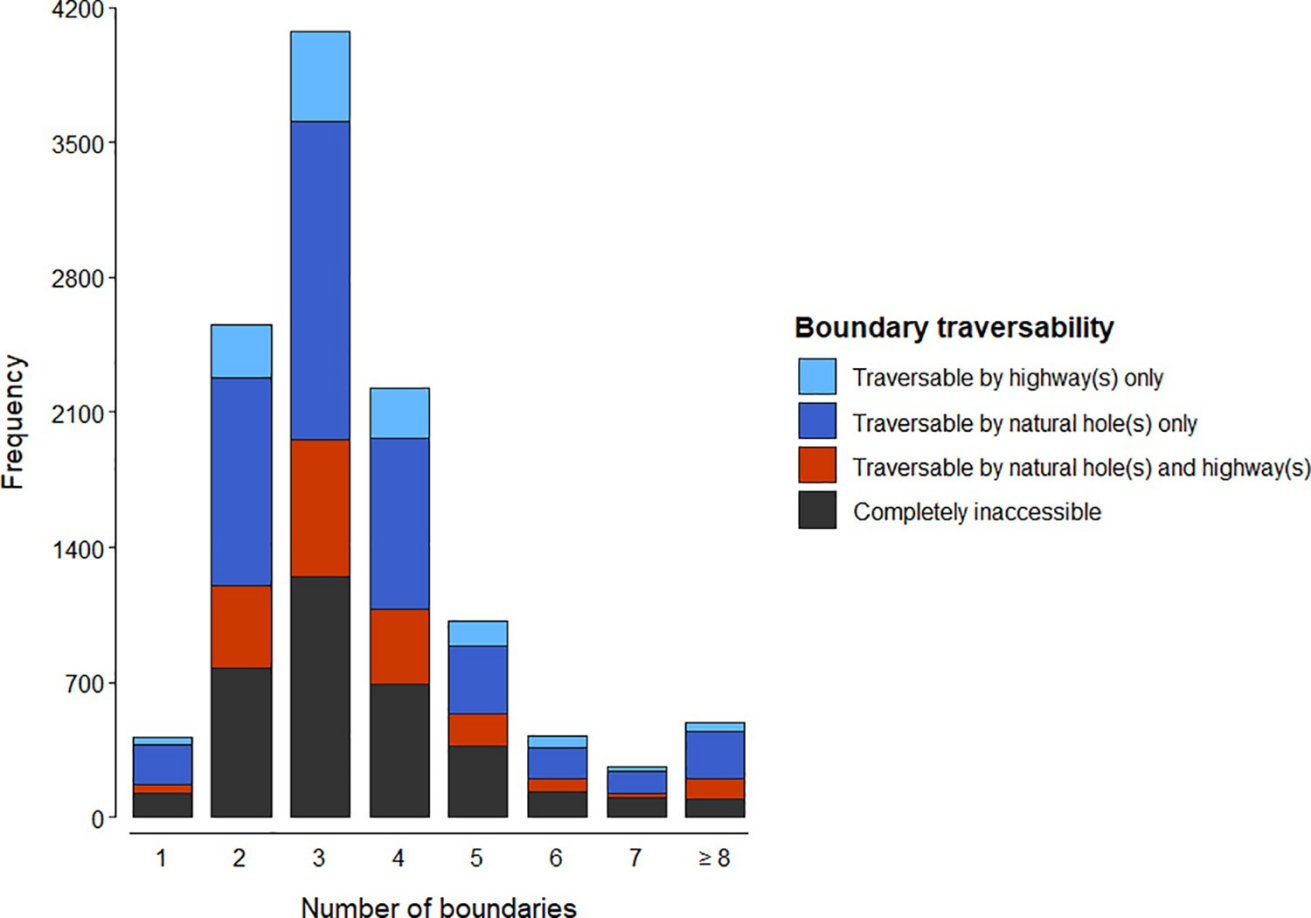
The number of back garden boundaries (N = 11,449) that could be traversed via hedgehog highways and / or naturally occurring holes in relation to the number of bordering gardens.

Collectively, the 3978 houses illustrated in Fig 1 were bordered by 11,449 boundaries (Fig 2). Of these boundaries, 3522 (30.8%) could not be traversed by hedgehogs at all; 4688 (40.9%), 1940 (16.9%) and 1308 (11.4%) were traversable via natural holes only, a combination of natural holes and hedgehog highways, and hedgehog highways only, respectively.

**Fig 3:**
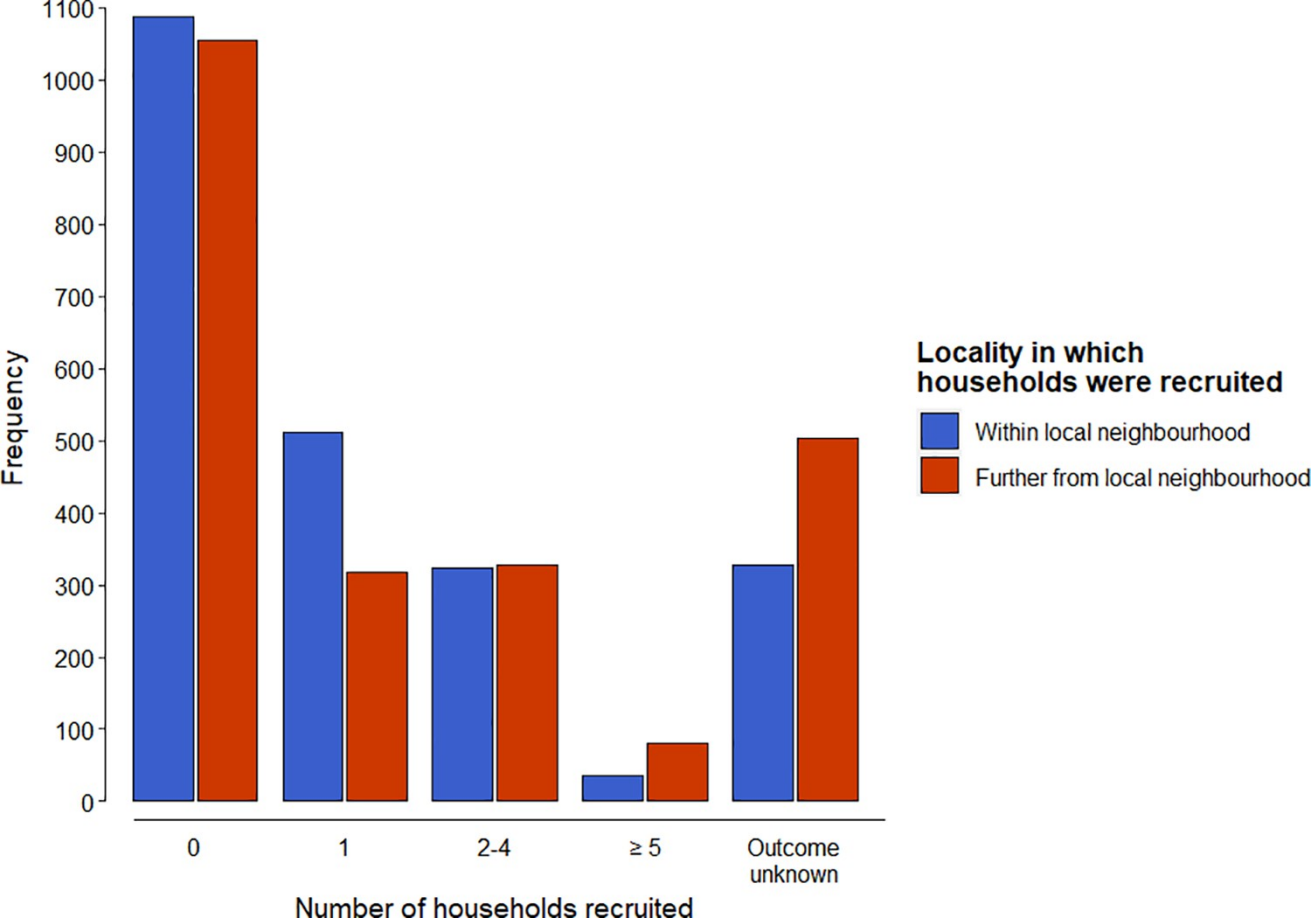
The number of additional households recruited to make hedgehog highways by Champions who had already made highways themselves (N = 2285) within their own neighbourhood and / or further afield.

The 2285 Champions in the 2020 survey who had successfully created at least one highway constructed a total of 4516 highways in their own gardens (1.98 per individual). Of these individuals, 1087 (47.6%) failed to recruit any further households into making a highway in their local neighbourhood, 511 (22.4%) recruited one additional household, 324 (14.2%) recruited 2–4 households, 35 (1.5%) recruited ≥5 households, and 328 (14.4%) attempted to recruit further households but were unaware of whether they had been successful (Fig 3). Comparable figures for areas beyond the local neighbourhood were 1055 (46.2%), 318 (13.9%), 327 (14.3%), 81 (3.5%) and 504 (22.1%), respectively. Assuming median values of 1, 3 and 6.5 for these three size classes, and assuming that respondents with unknown success had failed to generate any highways, these figures translate to a minimum of 1711 (0.75 per individual) additional hedgehog highways in the respondents’ immediate neighbourhood and 1826 (0.80 per individual) further afield. This would indicate that each successful Champion generated, on average, 3.53 highways.

However, 52.0% of Champions who responded to the 2020 survey had failed to create any highways at all. Incorporating these additional respondents, each Champion would have, on average, generated 1.69 highways each (0.95 in their own garden + 0.36 in their local neighbourhood + 0.38 further afield).

### Factors affecting the decision to have made a hedgehog highway

The inclusion (Table 3) and exclusion (Table 4) of the variables RODENT, FEEDHEDGEHOG and HEDGEHOG did not markedly affect coefficient values or model fit, although there were subtle differences. In both models, the decision to have made a hedgehog highway was significantly affected by YEARSRESIDED, HOUSETYPE, HOUSESETTING, GARDENFEATURES, BADGERFOX, HEDGEHOGSTREET, WATCHWILDLIFE and HOUSTETYPE*SETTING. In the model including RODENT, FEEDHEDGEHOG and HEDGEHOG, all three variables also had a significant effect (Table 3); the variable ENVIGROUPS was also retained to improve model fit. In the second model, the variables ENVIGROUPS, GARDENING and EMPLOYMENT were retained to improve model fit (Table 4).

**Table 3:** Summary of the binary logistic regression analysis examining the effects of garden- and householder-related variables on the respondent’s decision to make a hedgehog highway (HIGHWAY) (N = 5986).

	Estimate	Std. error	z value	Pr(>|z|)	Odds	95% CI	
(Intercept)	-3.6143	0.1768	-20.4404	<0.0001	0.0269	0.019–0.0379	
YEARSRESIDED (0–5 years)							
6–20 years	0.1949	0.0722	2.7008	0.0069	1.2152	1.055–1.3999	***
>21 years	0.1164	0.0772	1.5081	0.1315	1.1234	0.9658–1.3069	
HOUSETYPE (Detached)							
Semi-detached	0.3722	0.1034	3.5981	<0.0001	1.4509	1.1847–1.7772	***
Terraced	0.4830	0.1543	3.1309	0.0017	1.6210	1.1975–2.1935	**
Flat	0.7692	0.4460	1.7246	0.0846	2.1581	0.8898–5.2085	
HOUSESETTING (In a village or smaller)							
In a town or city	0.6362	0.0861	7.3865	<0.0001	1.8894	1.5964–2.2377	***
HOUSETYPE * HOUSESETTING (Detached house in village or smaller)							
Semi-detached house in a town or city	-0.4543	0.1312	-3.4626	0.0005	0.6349	0.4909–0.8211	***
Terraced house in a town or city	-0.4699	0.1808	-2.5991	0.0093	0.6251	0.4386–0.8912	**
Flat in a town or city	-1.0269	0.4998	-2.0545	0.0399	0.3581	0.1337–0.9612	*
GARDENFEATURES (Six or less)							
Seven or more	0.5047	0.0631	8.0037	<0.0001	1.6564	1.4641–1.8746	***
BADGERFOX (Not sighted)							
Sighted	-0.1239	0.0582	-2.1263	0.0335	0.8835	0.7881–0.9903	*
HEDGEHOG (Not sighted)							
Sighted	0.5621	0.1125	4.9977	<0.0001	1.7544	1.4091–2.1905	***
HEDGEHOGSTREET (Not aware)							
Aware	0.6738	0.1025	6.5718	<0.0001	1.9617	1.6076–2.4035	***
WATCHWILDLIFE (Less important or not important)							
Important or very important	0.5691	0.1018	5.5907	<0.0001	1.7666	1.4495–2.1607	***
ENVIGROUPS (Not a member)							
Is a member	-0.0790	0.0581	-1.3589	0.1742	0.9241	0.8245–1.0355	
FEEDHEDGEHOG (Not fed)							
Fed	0.9158	0.0879	10.4159	<0.0001	2.4988	2.1056–2.9724	***
RODENT (Not sighted)							
Sighted	0.2906	0.0878	3.3109	0.0009	1.3372	1.1265–1.5892	***

This analysis included the variables RODENT, FEEDHEDGEHOG and HEDGEHOG (see Methods). Reference levels for variables are indicated in parentheses. AIC = 7407.2; Hosmer and Lemeshow test: χ^2^_8_ = 7.83, p = 0.45; Nagelkerke *R*^2^ = 0.18.

* = p < 0.*05*

** = p < 0.*01*.

*** = p < 0.*001*.

**Table 4:** Summary of the binary logistic regression analysis examining the effects of garden and householder-related variables on the respondent’s decision to make a hedgehog highway (HIGHWAY) (N = 5986).

	Estimate	Std. error	z value	Pr(>|z|)	Odds	95% CI	
(Intercept)	-2.6388	0.1606	-16.4319	<0.0001	0.0714	0.052–0.0976	
YEARSRESIDED (0–5 years)							
6–20 years	0.3083	0.0700	4.4056	<0.0001	1.3611	1.1869–1.5615	***
>21 years	0.2529	0.0769	3.2862	0.0010	1.2877	1.1075–1.4975	**
HOUSETYPE (Detached)							
Semi-detached	0.4149	0.1026	4.0436	0.0001	1.5143	1.2384–1.8518	***
Terraced	0.4517	0.1505	3.0013	0.0027	1.5710	1.1688–2.1094	**
Flat	0.7781	0.4382	1.7757	0.0758	2.1773	0.9099–5.1672	
HOUSESETTING (In a village or smaller)							
In a town or city	0.6478	0.0848	7.6349	<0.0001	1.9112	1.619–2.2579	***
HOUSETYPE * HOUSESETTING (Detached house in village or smaller)							
Semi-detached house in a town or city	-0.5395	0.1291	-4.1784	<0.0001	0.5830	0.4526–0.7509	***
Terraced house in a town or city	-0.5371	0.1758	-3.0556	0.0022	0.5844	0.4142–0.8253	**
Flat in a town or city	-1.1986	0.4891	-2.4507	0.0143	0.3016	0.1151–0.7938	*
GARDENFEATURES (Six or less)							
Seven or more	0.6632	0.0620	10.6909	<0.0001	1.9410	1.7192–2.1926	***
BADGERFOX (Not sighted)							
Sighted	-0.1346	0.0567	-2.3748	0.0176	0.8741	0.7821–0.9767	*
HEDGEHOGSTREET (Not aware)							
Aware	0.9134	0.0994	9.1856	<0.0001	2.4928	2.0560–3.0368	***
WATCHWILDLIFE (Less important or not important)							
Important or very important	0.7295	0.0996	7.3261	<0.0001	2.0740	1.7095–2.5262	***
ENVIGROUPS (Not a member)							
Is a member	-0.0350	0.0570	-0.6144	0.5390	0.9656	0.8636–1.0797	
GARDENING (Less important or not important)							
Important or very important	-0.1061	0.0565	-1.8790	0.0602	0.8993	0.8050–1.0045	
EMPLOYMENT (Employed part-time)							
Employed full-time	0.0249	0.0799	0.3121	0.7550	1.0253	0.8767–1.1993	
Unemployed or homemaker	-0.0329	0.1270	-0.2594	0.7953	0.9676	0.7539–1.2404	
Student	-0.7055	0.2272	-3.1050	0.0019	0.4938	0.3112–0.7606	**
Retired	0.0283	0.0794	0.3562	0.7217	1.0287	0.8805–1.2019	
Prefer not to say / other	0.0790	0.1307	0.6040	0.5458	1.0822	0.8372–1.3980	

This analysis excluded the variables RODENT, FEEDHEDGEHOG and HEDGEHOG (see Methods). Reference levels for variables are indicated in parentheses. AIC = 7675.6; Hosmer and Lemeshow test: χ^2^_8_ = 10.52, p = 0.23; Nagelkerke *R*^2^ = 0.12.

* = p < 0.*05*

** = p < 0.*01*.

*** = p < 0.*001*.

**Fig 4:**
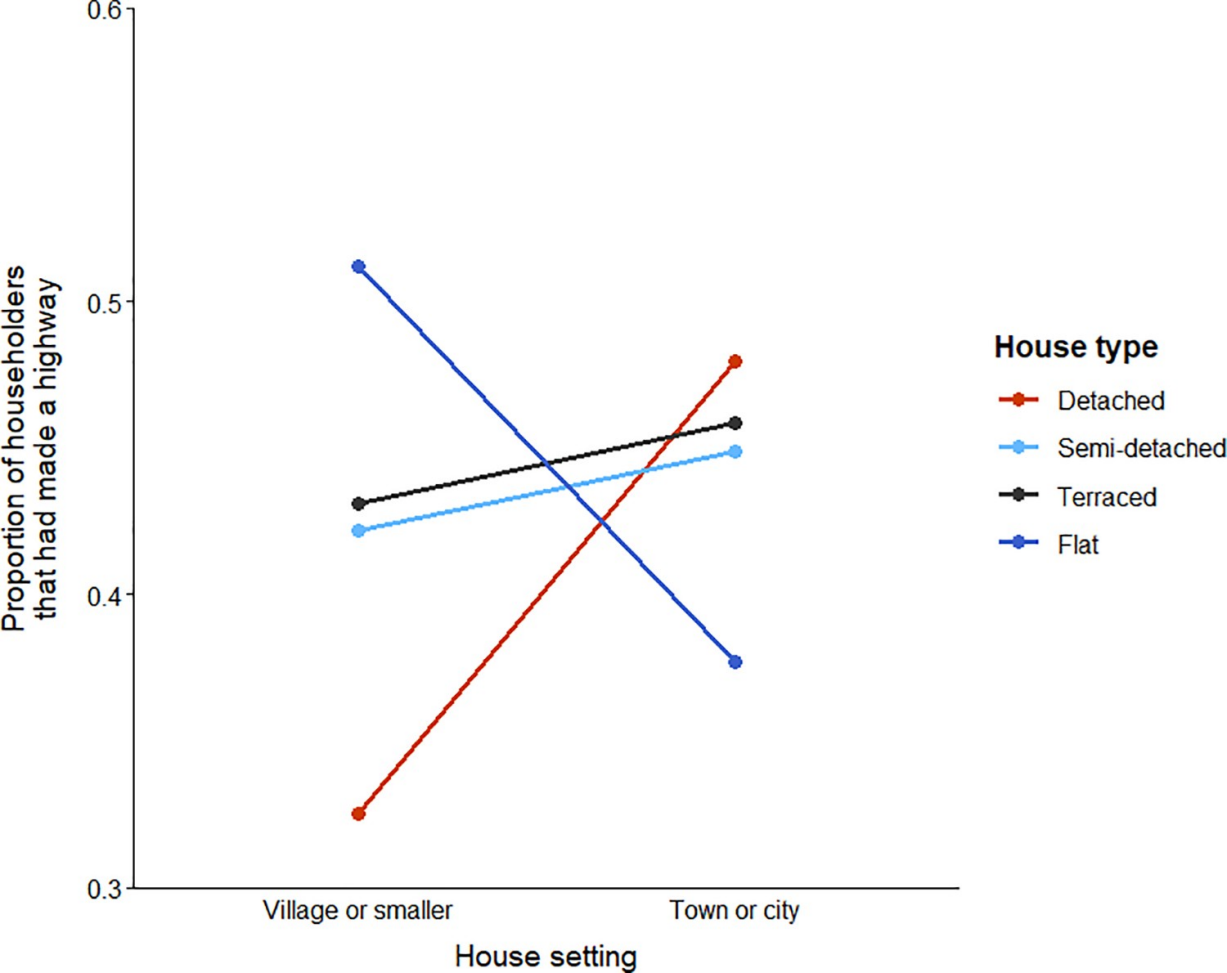
Probability that householders (N = 5986) had created a hedgehog highway in relation to house type and house location.

Focussing on the model outlined in Table 3, respondents were more likely to have created a highway if: they had lived in their home for 6–20 years; their garden contained ≥6 wildlife friendly features; they had sighted rodents or a hedgehog in their garden in the previous 12 months, but not a fox or badger; they were aware of the Hedgehog Street campaign; they ranked watching wildlife as important / very important; and they did feed hedgehogs. In general terms, respondents were significantly more likely to have created a highway if they lived in a semi-detached or terraced property, or lived within a town or city; however, respondents living in flats in a village or smaller hamlet were significantly more likely to have created a highway (Fig 4).

### Reasons cited for not having made a hedgehog highway

**Fig 5:**
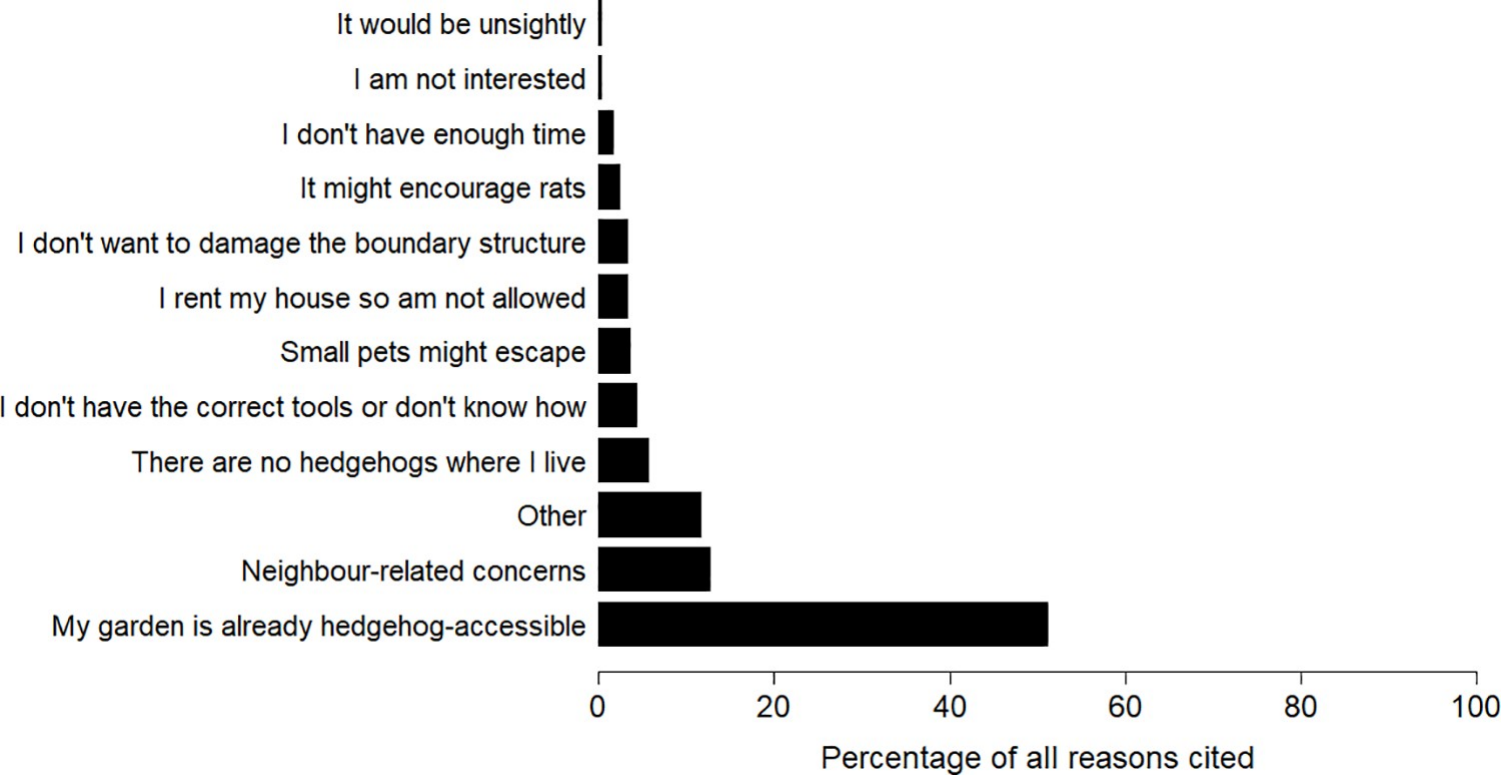
Reasons given by householders for not having created a hedgehog highway at the time of surveying. Figures are the percentage of all 4779 reasons cited by 3141 respondents.

Across all three surveys, 3362 respondents had not made a hedgehog highway; 3141 (93.4%) of these indicated why they had not done so, with a total of 4779 reasons given. The most common reason cited was that their garden was already accessible to hedgehogs (51.1%), followed by concerns relating to boundary ownership and / or talking to neighbours (12.6%; Fig 5).

## Discussion

Judging the success of the Hedgehog Street campaign is difficult because, as a public engagement exercise, there are few specific targets other than attempting to engage as many householders as possible, nor are there any quantified data on how frequently hedgehogs use these highways, although numerous photographs and videos online indicate that they are utilised readily once they are available. One potentially useful metric, however, is to estimate the proportion of Champions versus non-Champions that had created highways, and the number of highways created.

On average, the Hedgehog Champions that responded to the 2020 questionnaire survey generated a minimum of 1.69 highways each: 0.95 in their own garden, 0.36 in their immediate neighbourhood and 0.38 further afield. Extrapolating these figures to the total number of Champions enrolled at the time of surveying (N = 71,166), this would equate to the creation of >120,000 highways connecting >240,000 gardens, equivalent to approximately 1.1% of UK households with access to a garden [25]. These mean figures were, however, markedly reduced by the 52.0% of Champions who failed to create any highways; of the 48.0% of Champions that were successful, they led to the creation, on average, of 3.53 highways.

In comparison, significantly fewer respondents (6.1–29.8%) who stated that they had not heard of HS had created highways. Any estimate of the number of highways created nationally by these non-Champions is, however, dependent on the assumption that the householders in our study were a random sample of the UK population. Unfortunately, this does not appear to be the case: considering all respondents, significantly larger proportions of householders stated that they fed birds (81%), had created a pond (40%) and / or put up a bird box (68%) compared to the nationwide figures (51%, 16% and 21%, respectively) reported by Davies *et al*. [25]; respondents were also significantly more likely to have created a highway if they stated that watching wildlife was important to them. Moreover, a higher number of wildlife-friendly garden features within their garden increased the likelihood that respondents had made a highway by a factor of 1.66–1.94 (Tables 3, 4). This would suggest that our sample is dominated by householders who are innately more “wildlife-friendly” such that the 6.1–29.8% figures listed above are likely to be over-estimates. Nonetheless, since most UK householders are not registered as Champions, even a very low uptake rate (>0.4%) by non-Champions would result in the creation of a number of highways comparable to that which we have estimated for Champions.

The relatively low number of householders that have managed to successfully create highways compared to the numbers known to engage in other forms of wildlife-friendly activities [25] would suggest that: (i) there are significant obstacles to persuading householders and / or their neighbours to construct highways; and (ii) the benefits arising from their creation may be limited. The latter is associated with the impacts of highways on hedgehog movement trajectories and the increase in resources that are available in previously inaccessible gardens, which may, in turn, reduce the number of times roads need to be crossed. It is important, therefore, to consider how such changes might affect existing patterns of movement.

Approximately 83% of UK citizens live in urban areas [64], such that hedgehog highways are potentially most beneficial for urban hedgehog populations. In UK towns and cities, houses are frequently arranged in blocks consisting of two rows with rear gardens backing onto one another. To access the rear gardens of these houses, the preferred foraging habitat [42], hedgehogs can move from back garden to back garden and / or access the rear garden from the front via the side of the house where possible. Although there are numerous permutations of how adding even one highway could influence distances travelled, highways between neighbouring houses that are side-by-side could be associated with a reduction in the order of tens of metres, as a hedgehog would no longer need to leave one back garden to enter the other via the front of the second house. Conversely, a highway between two gardens that are back-to-back could result in a reduction in the order of a hundred metres or more, as the animal might not need to travel around the periphery of the block of houses to enter the second garden. Although these distances are small, Dowding *et al*. [42] recorded mean distances travelled of just 861m and 514m per night for male and female hedgehogs, respectively, in Bristol, UK, and Schaus Calderón [65] recorded comparable figures of 656m and 404m for hedgehogs in four urban sites across England. In this context, even the minor improvements in connectivity outlined above could be associated with reductions in nightly distances travelled of >10%; whether this would have a significant effect on the survival and / or reproductive output of hedgehogs is, however, unclear.

The biggest impact of highways would most likely be realised by enabling access to previously inaccessible gardens, as this would increase connectivity and potentially increase resource availability. Unfortunately, most highways in this study did not seem to increase connectivity in this way: 73.8% of Champions who had created a highway had done so after knowing that hedgehogs were visiting their garden, and 16.9% of boundaries between gardens were traversable by both a highway and a natural hole. In comparison, only 11.4% of garden boundaries and 3.2% of neighbouring gardens could be traversed and accessed, respectively, via a highway alone. Furthermore, the fact that hedgehogs were already visiting their garden was the most cited reason for respondents not having created a highway. Overall, only 5% of respondents thought their back garden was completely inaccessible to hedgehogs, although this may be an over-estimate as Williams *et al*. [66] reported a 70–80% discrepancy in the number of gardens considered inaccessible based on householder perceptions versus surveys performed by the researchers themselves. Nonetheless, these data do suggest that the fragmentation effect of garden boundaries in preventing access to gardens in the UK may not be as big a problem as has previously been supposed.

### Factors affecting the creation of hedgehog highways and future recommendations

In addition to increasing numbers of wildlife-friendly features and the importance that householders placed on watching wildlife, the decision to create a highway was: positively correlated with the householder’s length of occupation, house type, house location and whether the householder was aware of the Hedgehog Street campaign or not; but negatively associated with sightings of badgers and foxes. In addition, where they were included in the analyses, householders were more likely to have made a highway if they had seen hedgehogs and rodents in their garden, and if they fed hedgehogs. In many respects, these patterns reflect the reasons given by householders for not creating highways. For example, short periods of tenure are likely to be associated with people living in rental properties where landlords may prevent them changing the property’s boundaries. Similarly, although the creation of hedgehog highways could lead to hedgehogs being able to enter previously inaccessible gardens, 73.8% of respondents in the 2020 survey stated that they had created a highway after they knew hedgehogs were already visiting. As such, the positive relationship between highway construction and hedgehog presence in these analyses is most likely to be correlational rather than causal. This may also be the case for feeding hedgehogs (i.e. householders are likely to have started providing food once they knew hedgehogs were visiting their garden).

Likewise, the reduced likelihood that highways had been created in gardens where foxes and / or badgers had been sighted in the previous 12 months could reflect different underlying processes e.g. a conscious decision by householders to minimise the risk of predation, especially by badgers [32, 38, 67, 68], or hedgehogs are avoiding those gardens where foxes and badgers are present [46, 69]. However, it is important to note that, even in gardens where badgers are present, highways might allow hedgehogs to evade them more effectively and to access gardens that badgers cannot. Foxes, on the other hand, would likely be able to access all the same gardens as hedgehogs because of their greater agility, although the importance of foxes as a predator of hedgehogs is equivocal [70, 71]. In addition, there are numerous reports of hedgehogs visiting gardens in the presence of foxes and / or badgers with limited apparent conflict, although this is often associated with the provision of supplementary food; this food might, therefore, help to reduce predation risk but it is an extra level of involvement that not all members of the public would be willing to undertake. Consequently, on the balance of (albeit anecdotal) evidence, householders should not be discouraged from creating highways, even if they have sighted badgers or foxes in their garden.

Knowledge of the Hedgehog Street campaign was associated with the largest odds ratio values from the binary logistic regression analyses (Table 3), indicating that it was a particularly important factor associated with householders’ decisions to create a highway. Therefore, continuing to increase householders’ awareness of the potential benefits of creating highways is critical for the future expansion of this program. Further, based on the analyses of the results above, we make the following recommendations:

1. Additional effort needs to be focused on finding mechanisms to appeal to householders who are not inherently “wildlife-friendly”, as these comprise approximately half of all UK householders [25]. As such, these will often be the immediate neighbours of those householders who want to create highways and whose cooperation is therefore pivotal. The fact that 12.7% of householders stated that they had created a hedgehog highway but had not heard of Hedgehog Street does suggest that they had heard of the program’s underlying premise from some other source; these, and other, sources therefore need to be identified and expanded.
2. In parallel with the above, additional studies are required to help identify householders’ reservations concerning the creation of highways and to devise approaches for alleviating these concerns [see 72]. This will necessitate collaborations with social scientists [73] to devise multi-faceted approaches to help persuade householders with varying reasons for opposing the construction of hedgehog highways in their garden.
3. Greater emphasis needs to be placed on explaining how multiple highways from individual gardens would benefit hedgehogs. Householders should be advised that, even if hedgehogs are already accessing their garden, additional entry and exit points will help them move more efficiently through the wider landscape but these must be built into boundaries which hedgehogs cannot currently cross.
4. Additional data are required on the potential impact of predation by badgers and foxes on hedgehog populations in urban areas, and the patterns of interactions of these species in individual gardens.
5. Local planning authorities should commit to improving habitat connectivity for hedgehogs. The government’s National Planning Policy Framework for England [74] requires local plans to promote the conservation of priority species that are most threatened, which includes hedgehogs [75]. Developers therefore should be encouraged to incorporate hedgehog highways in property boundaries as standard, as has already been adopted by some companies [e.g.^76^, ^77^]. In addition, it is important to engage with owners of rental properties to facilitate the creation of highways in the one-third of UK homes that are rented [54].
6. Finally, field studies are required to quantify patterns of movement, energetic expenditure and hedgehog density in neighbourhoods before and after networks of highways have been constructed to identify the degree to which these affect individual animals and, ultimately, populations.

### Conclusion

Hedgehog Street has had significant success in recruiting participants and encouraging the creation of >120,000 highways by Hedgehog Champions. However, the fact that 52.0% of Champions surveyed had not been able to create a highway suggests that this initiative is impacted by challenges not normally evident in other public conservation campaigns; these include the need to interact with, and obtain permission from, immediate neighbours, the presence of hedgehogs in gardens leading to a perception that there is no need to create additional access points, and the creation of highways in boundaries that can already be traversed. Future studies therefore need to find mechanisms by which to address these limitations. Particular effort needs to be focused on identifying why householders are reluctant to create hedgehog highways so that strategies can be developed which address these concerns; such strategies must also target landlords and housing developers given the importance of rental properties in the UK and the current growth in housing construction. Finally, studies of hedgehog movement patterns are required so that the benefits of the creation of networks of hedgehog highways can be quantified.

## Supporting information

S1 File 2018 questionnaire. (DOCX)

S2 File 2019 questionnaire. (DOCX)

S3 File 2020 questionnaire. (DOCX)

S4 File Survey data. (XLSX)
