# Supplementary material for: An assessment of a conservation strategy to increase garden connectivity for hedgehogs that requires cooperation between immediate neighbours: A barrier too far?
Source: PLoS One. 2021 Nov 5;16(11):e0259537. doi: 10.1371/journal.pone.0259537 (PMC8570513; doi:10.1371/journal.pone.0259537)
Supplement: S1 File — (DOCX) [file pone.0259537.s001.docx]

2018 Questionnaire – text copy

*Introduction*

Thank you for agreeing to take part in this questionnaire survey. Please read the following information carefully. To comply with the Data Protection Act, you will be asked to give your consent before being forwarded to the questionnaire itself. This survey should take roughly 15 minutes to complete.

*Study aims*

The purpose of this project is to investigate people’s attitudes towards garden wildlife, and their willingness (or not) to carry out activities to help wildlife. As we are interested in getting a full range of opinions and attitudes, please consider taking part even if you are not particularly interested in wildlife.

*Confidentiality*

All information supplied will be treated in the strictest confidence, and there is no way that any individual taking part in the survey can be identified from the responses they give.

*Sensitive information requested*

As part of this survey we are requesting information on your age and your gender. For each of these questions, you will be given the option to not answer (“prefer not to say”) the question if you wish. However, we would like to reiterate that it will not be possible to identify any known individual from your responses to the questionnaire, and it would help us be able to perform a more detailed analysis if you did kindly answer these questions.

*Age restriction*

You must be at least 18 years old to take part in this survey.

*Country restrictions*

Because of the focus of this survey, you must live in the UK.

*Other restrictions*

Due to the nature of this survey and the information required, your property must have a garden in order for you to take part.

*Retention of information supplied*

It is hoped that the data collected will ultimately be published in a peer-reviewed scientific journal, but most immediately the data will be used by third-year undergraduate students at the University of Reading who are completing their Final Year Project as part of their degree programmes. The data collected will enable these students to investigate the concept of stakeholder engagement, which is a key component of successful wildlife management, as well as practice their analytical and critical evaluation skills.

*Consent*

By continuing with this survey, I conform that I have read the information supplied above. In particular, I am aware that:

(a) Some of the information requested can be considered personally sensitive, but I have the option to not answer these questions if I wish

(b) It will not be possible to identify me personally from any of the information I supply, including in any associated publications

(c) I must be 18 years or older, live in the UK and live in a property with access to a garden to complete this survey

(d) The data will be used by undergraduate students as part of their Final Year Project, but may also be submitted subsequently for publication in a scientific journal

1. **How old are you?**

18-24 25-30 31-40 41-50 51-60 61+ Prefer not to say

1. **What gender do you identify with?**

Male Female Other Prefer not to say

1. **What is your employment status?**

Work part time Work full time Unemployed Homemaker/stay at home parent Student Retired Prefer not to say

1. **Which county or region do you currently live in?**

Bedfordshire Berkshire Bristol Buckinghamshire Cambridgeshire Cheshire City of London Cornwall Cumbria Derbyshire Devon Dorset Durham East Riding of Yorkshire East Sussex Essex Gloucestershire Greater London Greater Manchester Hampshire Herefordshire Hertfordshire Isle of Wight Kent Lancashire Leicestershire Lincolnshire Merseyside Norfolk North Yorkshire Northamptonshire Northumberland Nottinghamshire Oxfordshire Rutland Shropshire Somerset South Yorkshire Staffordshire Suffolk Surrey Tyne and Wear Warwickshire West Midlands West Sussex West Yorkshire Wiltshire Worcestershire Wales Scotland Shetland Islands Isle of Man Jersey Guernsey

1. **How would you classify the position of your current home?**

Isolated In a small hamlet In a village In a town In a city

1. **What type of home do you live in?**

Detached house Semi-detached house End-terrace house Mid-terrace house Flat with access to a garden

1. **How many people live in your house?**

Number of adults (18+):

0 1 2 3 4 5+

Number of children (under 18):

0 1 2 3 4 5+

Number of temporary residents (e.g. children / stepchildren that may only be resident some of the time, students on holiday from University, etc.):

0 1 2 3 4 5+

1. **How long have you lived at your current address?**

Less than 1 year 1-5 years 6-10 years 11-20 years 21+ years

1. **Please indicate whether your property has the following (indicate more than one category if appropriate):**

Private front garden Private back garden Communal garden

1. **Please indicate whether you own or have previously owned the following** **pets**:

Dog(s):

Currently own Have previously owned Never owned

Cat(s):

Currently own Have previously owned Never owned

Caged pet(s) in the garden (e.g. rabbit or guinea pig):

Currently own Have previously owned Never owned

Chicken(s):

Currently own Have previously owned Never owned

Other (please specify): (open ended option)

1. **If you currently own a dog(s), how often do you let them outside into your garden?**

Several times a day They rarely go into the garden They never go into the garden N/A

1. **If you currently own a cat(s), how often do you let them outside?**

They are not allowed out day or night They are only allowed out during the day

They are only allowed out during the night They are allowed out both day and night N/A

1. **Are you a member of any environmental groups or charities? (E.g RSPB or National Trust)**

Yes No If yes, which are you a member of? (open ended option)

This part of the survey is aimed only at those who have a back garden. If you DO NOT have a back garden then please skip this section and go to Question 24 on the next page.

1. **How often do you spend time in your back garden each season?**

Spring:

Daily Few times a week Few times a month Less often than monthly Never

Summer:

Daily Few times a week Few times a month Less often than monthly Never

Autumn:

Daily Few times a week Few times a month Less often than monthly Never

Winter:

Daily Few times a week Few times a month Less often than monthly Never

1. **Please indicate how important the following are, in terms of what activities you carry out in your back garden:**

Watching birds:

Very important Important A little bit important Not important

Watching other wildlife:

Very important Important A little bit important Not important

Socialising:

Very important Important A little bit important Not important

Gardening:

Very important Important A little bit important Not important

Growing fruit and/or vegetables:

Very important Important A little bit important Not important

Relaxing:

Very important Important A little bit important Not important

Exercising/sports:

Very important Important A little bit important Not important

Use by children:

Very important Important A little bit important Not important

Use by pets:

Very important Important A little bit important Not important

Hanging washing out:

Very important Important A little bit important Not important

Storage:

Very important Important A little bit important Not important

1. **How many gardens does your back garden border?**

0 1 2 3 4+

1. **Of these gardens, how many do you think a terrestrial animal such as a hedgehog could access from your own garden (e.g. through a hole in a fence or a hole under the fence)?**

0 1 2 3 4+

1. **Do you think your back garden is accessible from your front garden for a terrestrial animal such as a hedgehog (e.g. under a gate or simply down the side of your house)?**

Yes No N/A

1. **What types of boundaries do you have around your garden? Please tick all that apply.**

Wooden fence Concrete / brick wall Wire fence Hedge Other (please specify) (open ended option)

1. **How often do you feed the following animals in your garden?**

Birds on a bird table or feeder:

Frequently Sometimes Never

Birds on the ground:

Frequently Sometimes Never

Birds of prey:

Frequently Sometimes Never

Hedgehog:

Frequently Sometimes Never

Badger:

Frequently Sometimes Never

Fox:

Frequently Sometimes Never

1. **Please indicate how often you have seen, or found signs of, the following animal species in your back garden over the course of the last 12 months.**

Birds:

Daily Few times a week Few times a month Less than monthly Never

Bats:

Daily Few times a week Few times a month Less than monthly Never

Deer:

Daily Few times a week Few times a month Less than monthly Never

Badgers:

Daily Few times a week Few times a month Less than monthly Never

Foxes:

Daily Few times a week Few times a month Less than monthly Never

Hedgehogs:

Daily Few times a week Few times a month Less than monthly Never

Squirrels:

Daily Few times a week Few times a month Less than monthly Never

Butterflies/bees/dragonflies, etc.:

Daily Few times a week Few times a month Less than monthly Never

Rats and mice:

Daily Few times a week Few times a month Less than monthly Never

1. **For each of the following wildlife-friendly garden features, please indicate whether you currently have them in your back garden, or if you would consider having them in the future.**

Grass-free flowering lawn:

Currently have Would consider having in the future Would not consider having

Hedges:

Currently have Would consider having in the future Would not consider having

Wilderness patch/wildflowers:

Currently have Would consider having in the future Would not consider having

Log pile:

Currently have Would consider having in the future Would not consider having

Pond:

Currently have Would consider having in the future Would not consider having

Drinking water for animals excluding birds (e.g. shallow dish for hedgehogs):

Currently have Would consider having in the future Would not consider having

Bird bath:

Currently have Would consider having in the future Would not consider having

Bird table:

Currently have Would consider having in the future Would not consider having

Bird box:

Currently have Would consider having in the future Would not consider having

Bat box:

Currently have Would consider having in the future Would not consider having

Hedgehog house:

Currently have Would consider having in the future Would not consider having

Insect hotel:

Currently have Would consider having in the future Would not consider having

Compost heap/bin:

Currently have Would consider having in the future Would not consider having

Hedgehogs are in serious decline in the UK, but are not uncommon in urban areas. However, one challenge they face in urban areas is getting into gardens and moving easily between them. Therefore, one way to help hedgehogs is to create a “hedgehog highway”: this can be hole in your fence or a hole under your fence. Examples of these hedgehog highways are illustrated in the photographs below. This is the focus of the People’s Trust for Endangered Species / British Hedgehog Preservation Society’s “Hedgehog Street” campaign (see www.hedgehogstreet.org)

1. **Prior to this questionnaire survey, had you heard about the Hedgehog Street campaign?**

Yes No

1. **Have you made a hedgehog highway (a hole in your fence, or a hole under your fence) to make your garden more accessible to hedgehogs?**

Yes No

1. **Earlier in this questionnaire, you indicated how many other back gardens you considered a hedgehog would be able to access from your own garden. How many of these other gardens can be accessed ONLY because you created a hedgehog highway?**

0 1 2 3 4+ N/A I have not made any hedgehog highways N/A I did not answer the earlier question as I do not have a back garden

The following 3 questions are for those people who have not made a hedgehog highway in or under any of their garden boundaries.

If you have made a hedgehog highway in your garden then please skip to Question 29 on the next page.

1. **Why have you not done anything to improve your gardens accessibility for hedgehogs (e.g. cut holes in or under fences)? Please tick all that apply.**

There are no hedgehogs where I live

There are already enough hedgehogs in my area

I am not interested in hedgehogs

I rent my house so am not allowed

It could lower the value of my house, if I wanted to sell

My garden is already accessible to hedgehogs

It might encourage rats

Small pets might escape

My neighbour owns the fences

I don't want to talk with my neighbour

I don't think my neighbour would allow it

I don't want to damage the boundary structure

It would be unsightly

I was not aware that this would help hedgehogs

I don't know how to do this

I don't have enough time

I don't have the right tools to do this

Other (please specify)

1. **After now hearing about the hedgehog street, how likely would you be to create a 'hedgehog highway' in the next 6 months?**

Definitely not Unlikely Maybe, but not a priority Likely Definitely

1. **Given that increasing access to and connectivity between gardens is a priority for the conservation of urban hedgehogs, but that many homeowners have reasons for not building hedgehog highways, we are interested in hearing what hedgehog conservationists might be able to do to encourage you to help. Any suggestions are welcome!**
2. **Imagine we are about to have a General Election in a couple of weeks time, and the major political parties have been campaigning on a wide range of issues. One of the issues they have been discussing is the environment (e.g. the conservation of wildlife, ensuring that nature reserves are protected, making sure that animals are not killed illegally). In the context of your vote, how important would the environmental issues be in influencing how you voted?**

Very important Important A little bit important Not important

1. **Please indicate whether you agree or disagree with each of the following statements:**

Seeing wildlife enriches my life and has a positive effect on my well-being:

Strongly disagree Disagree No opinion Agree Strongly agree Unsure

It is important to conserve wildlife (animals/plants) in the countryside:

Strongly disagree Disagree No opinion Agree Strongly agree Unsure

It is important to conserve wildlife (animals/plants) in towns and cities:

Strongly disagree Disagree No opinion Agree Strongly agree Unsure

Urban residents should be encouraged to help wild animals by putting out food:

Strongly disagree Disagree No opinion Agree Strongly agree Unsure

Urban residents should be encouraged to help hedgehogs by cutting holes in their fences:

Strongly disagree Disagree No opinion Agree Strongly agree Unsure

Farmers should be encouraged to re-plant hedgerows in their fields:

Strongly disagree Disagree No opinion Agree Strongly agree Unsure

Farmers should be encouraged to use fewer chemicals:

Strongly disagree Disagree No opinion Agree Strongly agree Unsure

Farmers should be allowed to kill badgers to prevent the spread of bovine tuberculosis:

Strongly disagree Disagree No opinion Agree Strongly agree Unsure

Farmers should be allowed to kill badgers to protect hedgehogs:

Strongly disagree Disagree No opinion Agree Strongly agree Unsure

Farmers should be allowed to kill foxes to protect wild animals and birds:

Strongly disagree Disagree No opinion Agree Strongly agree Unsure

End of survey.
