# Supplementary material for: An assessment of a conservation strategy to increase garden connectivity for hedgehogs that requires cooperation between immediate neighbours: A barrier too far?
Source: PLoS One. 2021 Nov 5;16(11):e0259537. doi: 10.1371/journal.pone.0259537 (PMC8570513; doi:10.1371/journal.pone.0259537)
Supplement: S2 File — (DOCX) [file pone.0259537.s002.docx]

2019 Questionnaire – text copy

**Consent**

*Introduction*

Thank you for agreeing to take part in this questionnaire survey. Please read the following information carefully. To comply with the Data Protection Act, you will be asked to give your consent before being forwarded to the questionnaire itself. This survey should take roughly 15 minutes to complete.

*Study aims*

The purpose of this project is to investigate people’s attitudes towards garden wildlife, and their willingness (or not) to carry out activities to help wildlife. As we are interested in getting a full range of opinions and attitudes, please consider taking part even if you are not particularly interested in wildlife.

*Confidentiality*

All information supplied will be treated in the strictest confidence, and there is no way that any individual taking part in the survey can be identified from the responses they give.

*Sensitive information requested*

As part of this survey, we are requesting information on your age and your gender. For each of these questions, you will be given the option to not answer (“prefer not to say”) if you wish. However, we would like to reiterate that it will not be possible to identify any known individual from their responses to the questionnaire, and it would help us be able to perform a more detailed analysis if you did kindly answer these questions.

*Age restriction*

You must be at least 18 years old to take part in this survey.

*Country restrictions*

Because of the focus of this survey, you must live in the UK.

*Other restrictions*

Due to the nature of this survey, and the information required, your house must have a garden in order for you to take part.

*Retention of information supplied*

It is hoped that the data collected will ultimately be published in a peer-reviewed scientific journal, but most immediately the data will be used by third-year undergraduate students at the University of Reading who are completing their Final Year Project as part of their degree programmes. The data collected will enable these students to investigate the concept of stakeholder engagement, which is a key component of successful wildlife management, as well as practice their analytical and critical evaluation skills.

*Definitions*

At several points in this survey we will supply definitions for important terms that we use in some of the questions. Please make sure that you familiarize yourself with these definitions so that you answer all of the questions consistently.

*Consent*

By continuing with this survey, I conform that I have read the information supplied above. In particular, I am aware that:

(a) Some of the information requested can be considered personally sensitive, but I have the option to not answer these questions if I wish

(b) It will not be possible to identify me personally from any of the information I supply, including in any associated publications

(c) I must be 18 years or older, live in the UK and live in a property with access to a garden to complete this survey

(d) The data will be used by undergraduate students as part of their Final Year Project, but may also be submitted subsequently for publication in a scientific journal

Agree (Please tick this box to continue to the questions)

1. **How old are you?**

18-24 25-30 31-40 41-50 51-60 61+ Prefer not to say

1. **What gender do you identify with?**

Male Female Other Prefer not to say

1. **What is your employment status?**

Work part time Work full time Unemployed Homemaker/stay at home parent Student Retired Prefer not to say

1. **Which county or region do you currently live in?**

Bedfordshire Berkshire Bristol Buckinghamshire Cambridgeshire Cheshire City of London Cornwall Cumbria Derbyshire Devon Dorset Durham East Riding of Yorkshire East Sussex Essex Gloucestershire Greater London Greater Manchester Hampshire Herefordshire Hertfordshire Isle of Wight Kent Lancashire Leicestershire Lincolnshire Merseyside Norfolk North Yorkshire Northamptonshire Northumberland Nottinghamshire Oxfordshire Rutland Shropshire Somerset South Yorkshire Staffordshire Suffolk Surrey Tyne and Wear Warwickshire West Midlands West Sussex West Yorkshire Wiltshire Worcestershire Wales Scotland Shetland Islands Isle of Man Jersey Guernsey

1. **What is your nationality? If you do not wish to say, please type "Prefer not to say" in the answer box.**
2. **How would you classify the position of your current home?**

Isolated In a small hamlet In a village In a town In a city

1. **What type of home do you live in?**

Detached house Semi-detached house End-terrace house Mid-terrace house Flat with access to a garden

1. **How many people live in your house?**

Number of adults (18+):

Number of children (under 18):

Number of temporary residents (e.g. children / stepchildren that may only be resident some of the time, students on holiday from University, etc.):

1. What is your employment status?

Work part time Work full time Unemployed Homemaker Full-time student Student but also work part time Retired Prefer not to say

1. **Please indicate your occupation. For this question we are using the same divisions as outlined by the Office of National Statistics (ONS). The ONS recognises the following 9 divisions**

Managers, directors and senior officials Professional occupation Associate professional and technical occupations Administrative and secretarial occupations Skilled trades occupation Caring, leisure and other service occupation Process, plant and machine operatives Elementary occupations N/A

1. **If you are living in your home with a partner, what is their employment status?**

Work part time Work full time Unemployed Homemaker Full-time student Student but also work part time Retired Prefer not to say

1. **How long have you lived at your current address?**

Less than 1 year 1-5 years 6-10 years 11-20 years 21+ years

1. **Do you rent or own your current house?**

Rent Own

1. **Please indicate whether your property has the following (indicate more than one category if appropriate):**

Private front garden Private back garden Communal garden

1. **Please indicate whether you own or have previously owned the following** **pets:**

Dog(s):

Currently own Have previously owned Never owned

Cat(s):

Currently own Have previously owned Never owned

Caged pet(s) in the garden (e.g. rabbit or guinea pig):

Currently own Have previously owned Never owned

Chicken(s):

Currently own Have previously owned Never owned

Other (please specify): (open ended option)

1. **Are you a member of any environmental groups or charities? (E.g RSPB or National Trust)**

Yes No If yes, which are you a member of? (open ended option)

1. **In their most recent TV series, the BBC Springwatch programme (in conjunction with the British Trust for Ornithology and the Open University) ran their GardenWatch citizen science project. In this project, viewers were asked to complete up to four different tasks/surveys, each with a slightly different focus:**

**(i) Beyond the back door – what are your garden’s defining features?**

**(ii) Bird detective – what are our garden birds getting up to?**

**(iii) Mammal detective – who are our furry neighbours?**

**(iv) Worm detective – what’s living below ground?**

**Did you participate in any of these four projects?**

(i) Beyond the back door

Yes No

(ii) Bird detective

Yes No

(iii) Mammal detective

Yes No

(iv) Worm detective

Yes No

This part of the survey is aimed only at those who have a back garden. If you DO NOT have a back garden then please skip Questions 18 to 24 and jump straight to Question 25.

1. **Please indicate how important the following are, in terms of what activities you carry out in your back garden:**

Watching birds:

Very important Important A little bit important Not important

Watching other wildlife:

Very important Important A little bit important Not important

Socialising:

Very important Important A little bit important Not important

Gardening:

Very important Important A little bit important Not important

Growing fruit and/or vegetables:

Very important Important A little bit important Not important

Relaxing:

Very important Important A little bit important Not important

Exercising/sports:

Very important Important A little bit important Not important

Use by children:

Very important Important A little bit important Not important

Use by pets:

Very important Important A little bit important Not important

Hanging washing out:

Very important Important A little bit important Not important

Storage:

Very important Important A little bit important Not important

1. **How often do you feed the following animals in your garden?**

Badger:

Frequently Sometimes Never

Garden birds on the ground:

Frequently Sometimes Never

Garden birds on a table or from a feeder:

Frequently Sometimes Never

Hedgehog:

Frequently Sometimes Never

Fox:

Frequently Sometimes Never

1. **What is the most common type of boundary around your back garden? Please tick one option.**

Wooden fence Concrete/brick wall Wire fence Hedge Other (please specify)

1. **If you answered “wooden fence” to the previous question, we would like to know what sort of fence you have based upon its structure at ground level. Please answer "not applicable" if your answer to the previous question was not "wooden fence".**

Vertical slats that go right to the ground

Fence panel/vertical slats with a horizontal wooden gravel board at ground level

Fence panel/vertical slats with a horizontal concrete gravel board at ground level

Other type of wooden fence

Not applicable

1. **Excluding the most common type of boundary which you indicated in Question 19, what other type of boundaries do you around your back garden? Please tick more than one option if applicable.**

Wooden fence Concrete/brick wall Wire fence Hedge Other (please specify)

1. **For each of the following wildlife-friendly garden features, please indicate whether you currently have them in your back garden, or if you would consider having them in the future.**

Grass-free flowering lawn:

Currently have Would consider having in the future Would not consider having

Hedges:

Currently have Would consider having in the future Would not consider having

Wilderness patch/wildflowers:

Currently have Would consider having in the future Would not consider having

Log pile:

Currently have Would consider having in the future Would not consider having

Pond:

Currently have Would consider having in the future Would not consider having

Drinking water for animals excluding birds (e.g. shallow dish for hedgehogs):

Currently have Would consider having in the future Would not consider having

Bird bath:

Currently have Would consider having in the future Would not consider having

Bird table:

Currently have Would consider having in the future Would not consider having

Bird box:

Currently have Would consider having in the future Would not consider having

Bat box:

Currently have Would consider having in the future Would not consider having

Hedgehog house:

Currently have Would consider having in the future Would not consider having

Insect hotel:

Currently have Would consider having in the future Would not consider having

Compost heap/bin:

Currently have Would consider having in the future Would not consider having

1. **Please indicate how often you have seen, or found signs of, the following animal species in your back garden over the course of the last 12 months.**

Badgers:

Daily Few times a week Few times a month Less than monthly Never

Foxes:

Daily Few times a week Few times a month Less than monthly Never

Hedgehogs:

Daily Few times a week Few times a month Less than monthly Never

Rats and mice:

Daily Few times a week Few times a month Less than monthly Never

1. **Please indicate whether you think each of the following species has increased in abundance, decreased in abundance or stayed roughly the same over the course of the last 5 years in your local neighbourhood. If you have not lived in your house for five years or more, please answer "Not lived here long enough". Please tick one option in each row.**

Badgers:

Decreased Stayed the same Increased Not lived here long enough

Foxes:

Decreased Stayed the same Increased Not lived here long enough

Hedgehogs:

Decreased Stayed the same Increased Not lived here long enough

Rats:

Decreased Stayed the same Increased Not lived here long enough

Hedgehogs are in serious decline in the UK, but can be commonly found in gardens in cities, towns, villages and rural areas. However, one challenge they do face is accessing all the gardens in an area, and moving easily between them. Therefore, one way you can help hedgehogs is to create a "hedgehog highway": this can be a hole in your fence or a hole under your fence. Examples of these hedgehog highways are illustrated in the photographs below. This is the focus of the People's Trust for Endangered Species/British Hedgehog Preservation Society's "Hedgehog Street" campaign (see www.hedgehogstreet.org).

1. **Prior to this survey, had you heard about the Hedgehog Street campaign?**

Yes No

1. **As part of their Hedgehog Street campaign, members of the public can sign up to be a Hedgehog Champion. When you register as a Hedgehog Champion you are then given access to a wide range of resources that would help you tell your friends and neighbours about hedgehogs and how you can help them (e.g. slides, fact sheet, invitation card, poster, top tips factsheet). Have you signed up to become a Hedgehog Champion?**

Yes No

More about hedgehog highways

The following questions are designed to investigate how easy it is for hedgehogs to move into and out of your garden(s). In particular, we are interested in how they can enter or leave your garden as a consequence of (a) naturally occurring holes versus (b) hedgehog highways. For the purposes of these questions, we define naturally occurring holes as "holes through or under you garden borders (e.g. fences) which you did not personally create". These could have arisen in a number of different ways including, for example, as a consequence of an animal digging under your fence or pushing through a hedge, the type of fencing in your garden (e.g. not all fencing goes all the way to the ground), and / or general wear and tear of your fencing. In contrast, hedgehog highways are defined as "any hole through or under you garden borders (e.g. fences) which was DELIBERATELY created for the purposes of helping hedgehogs get into or out of your garden". These hedgehog highways may have been created by you personally, or your neighbour(s).

1. **Please indicate the answer that best describes whether a hedgehog can or cannot access your back garden from your front garden.**

Yes, a hedgehog can access my back garden from my front garden ONLY through a naturally occurring hole (e.g. under a gate, down the side of the house)

Yes, a hedgehog can access my back garden from my front garden but ONLY through a hedgehog highway

Yes, a hedgehog can access my back garden from my front garden through BOTH a naturally occurring hole and a hedgehog highway

No, a hedgehog cannot access my back garden from my front garden

Not applicable (e.g. I live in a mid-terraced house, I don't have a back garden)

1. **How many other back gardens border your own back garden (this includes gardens on either side of your garden and/or at the back of your garden)? Please remember this number, as it is important for the next three questions.**

1 2 3 4 5 6 7 8 9 10 More than 10

1. **Of those gardens indicated in Question 29, how many can a hedgehog access from your own garden ONLY by a natural hole?**

1 2 3 4 5 6 7 8 9 10 More than 10

1. **Of those gardens indicated in Question 29, how many can a hedgehog access from your own garden ONLY via a hedgehog highway deliberately made by you or your neighbour?**

1 2 3 4 5 6 7 8 9 10 More than 10

1. **Of those gardens indicated in Question 29, how many can a hedgehog access from your own garden BOTH via a natural hole and a hedgehog highway?**

1 2 3 4 5 6 7 8 9 10 More than 10

A quick check before you move on

Please make sure that the number of gardens you have indicated in Questions 30, 31 and 32 add up to the total number of gardens indicated in Question 29.

1. **Have you made a hedgehog highway (remember this is a hole in your fence or under your fence that you have created deliberately) to make your back garden more accessible to hedgehogs?**

Yes, I have made a hedgehog highway

No, I have not made a hedgehog highway myself, but my neighbour has made one in/under our shared fence

No, I have not made a hedgehog highway myself

If you answered "No" to the previous question, please answer the next three questions (Questions 34, 35 and 36) which are for people that have NOT PERSONALLY CREATED A HEDGEHOG HIGHWAY. Once you have answered these three questions, please skip forward to Question 39.

1. **After hearing about Hedgehog Street, how likely would you be to create a hedgehog highway in the next 12 months?**

Definitely not Unlikely Maybe, but not a priority Likely Definitely

1. **Why have you not done anything to improve your gardens accessibility for hedgehogs (e.g. cut holes in or under fences)? Please tick all that apply.**

There are no hedgehogs where I live

There are already enough hedgehogs in my area

I am not interested in hedgehogs

I rent my house so am not allowed

It could lower the value of my house, if I wanted to sell

My garden is already accessible to hedgehogs

It might encourage rats

Small pets might escape

My neighbour owns the fences

I don't want to talk with my neighbour

I don't think my neighbour would allow it

I don't want to damage the boundary structure

It would be unsightly

I was not aware that this would help hedgehogs

I don't know how to do this

I don't have enough time

I don't have the right tools to do this

Other (please specify)

1. **Given that increasing connectivity between gardens is a priority for the conservation of urban hedgehogs, but that many homeowners have reasons for not building hedgehog highways, we are interested in hearing what hedgehog conservationists might be able to do to encourage you to help. Any suggestions welcome!**
2. **This question (and the next question) are for people that have personally created a hedgehog highway. If you have not personally created a hedgehog highway, please skip forward to Question 39. In total, how many hedgehog highways have you created in your back garden?**
3. **Did you create your hedgehog highway(s) (a) knowing that hedgehogs were already visiting your garden or (b) without knowing whether hedgehogs were or were not visiting your garden?**

I created my hedgehog highway(s) knowing that hedgehogs were already visiting my garden

I created hedgehog highway(s) without knowing whether hedgehogs were or were not already visiting my garden

1. **Please indicate whether you agree or disagree with each of the following statements:**

Seeing wildlife enriches my life and has a positive effect on my well-being:

Strongly disagree Disagree No opinion Agree Strongly agree

It is important to conserve wildlife (animals/plants) in the countryside:

Strongly disagree Disagree No opinion Agree Strongly agree

It is important to conserve wildlife (animals/plants) in towns and cities:

Strongly disagree Disagree No opinion Agree Strongly agree

Urban residents should be encouraged to help wild animals by putting out food:

Strongly disagree Disagree No opinion Agree Strongly agree

Urban residents should be encouraged to help hedgehogs by cutting holes in their fences:

Strongly disagree Disagree No opinion Agree Strongly agree

Farmers should be encouraged to re-plant hedgerows in their fields:

Strongly disagree Disagree No opinion Agree Strongly agree

Farmers should be encouraged to use fewer chemicals:

Strongly disagree Disagree No opinion Agree Strongly agree

Householders should be encouraged to use fewer chemicals in their gardens:

Strongly disagree Disagree No opinion Agree Strongly agree

Farmers should be encouraged to plant more hedges:

Strongly disagree Disagree No opinion Agree Strongly agree

Householders should be encouraged to make their gardens more wildlife friendly:

Strongly disagree Disagree No opinion Agree Strongly agree

It is okay to kill badgers to protect hedgehogs:

Strongly disagree Disagree No opinion Agree Strongly agree

It is okay to kill foxes to protect hedgehogs:

Strongly disagree Disagree No opinion Agree Strongly agree

Hedgehogs belong in the countryside more than towns and cities:

Strongly disagree Disagree No opinion Agree Strongly agree

Wildlife gardens look too messy:

Strongly disagree Disagree No opinion Agree Strongly agree

I would be more prepared to create a wildlife garden if I knew what plants to plant:

Strongly disagree Disagree No opinion Agree Strongly agree

I keep my garden neat and tidy because that is what I prefer:

Strongly disagree Disagree No opinion Agree Strongly agree

I keep my garden neat and tidy because that is what I think my neighbours expect:

Strongly disagree Disagree No opinion Agree Strongly agree

I would be more willing to create a hedgehog highway in my fence if I knew lots of my neighbours had already done so:

Strongly disagree Disagree No opinion Agree Strongly agree

Most urban wildlife are pests:

Strongly disagree Disagree No opinion Agree Strongly agree

Cutting a hole in my fence is tantamount to vandalism:

Strongly disagree Disagree No opinion Agree Strongly agree

I am not concerned about the plight of hedgehogs:

Strongly disagree Disagree No opinion Agree Strongly agree

I have too many other concerns in my life to worry about hedgehogs:

Strongly disagree Disagree No opinion Agree Strongly agree

End of survey.
