# Supplementary material for: An assessment of a conservation strategy to increase garden connectivity for hedgehogs that requires cooperation between immediate neighbours: A barrier too far?
Source: PLoS One. 2021 Nov 5;16(11):e0259537. doi: 10.1371/journal.pone.0259537 (PMC8570513; doi:10.1371/journal.pone.0259537)
Supplement: S3 File — (DOCX) [file pone.0259537.s003.docx]

2020 Questionnaire – text copy

Hedgehog Champion survey

Thank you for your interest in our survey. We’d like to see how effective the Hedgehog Street campaign has been at making positive impacts for hedgehogs in gardens and local communities. The data you will provide is vital to assess the impact of this important project. 

You’ll be asked a few details about the garden of your **current**home and how you use it. Then we’d like to know whether you or your neighbours have been able to make any Hedgehog Highways. Finally, we have some questions about you and where you live. The survey will take around 10 minutes.

**Even if you don’t have hedgehogs or any links into your garden, it would still be fantastic to hear from you so that we can get as full a picture as possible of hedgehogs in the UK.**

**Please only complete this survey if you live in a property with access to a garden.** This survey is for residents of the UK who are at least 18 years old. All information supplied will be treated in the strictest confidence. The data collected will be analysed by a postgraduate student at the University of Reading as part of her PhD studies, and to further develop the Hedgehog Street campaign.

**Consent:**By continuing with this survey, I confirm that I have read the information above and am aware that it will not be possible to identify me personally from any of the information I supply; I am 18 years or older; live in mainland England, Scotland or Wales; live in a property with access to a garden; and that the data will be used by a student at Reading University for her PhD, and may also be submitted subsequently for publication in a scientific journal.

**Part 1 – questions about your back garden**

We would like to know a bit about your back garden. It will tell us what factors, other than Hedgehog Highways, might affect hedgehog presence in your garden.

2. You have been contacted for this survey because you enrolled as a Hedgehog Champion through the Hedgehog Street campaign, which launched in 2011. When did you sign up to become a Hedgehog Champion?

- In the last month
- In the last year
- In the last 2-3 years
- In the last 4-5 years
- More than 5 years ago
- I don’t remember
- I am not a Hedgehog Champion

3. Please indicate how important the following are to you, in terms of activities you carry out in your back garden:

*Please select one option from each row.*

|  | Very important | Important | Somewhat important | Not important |
| --- | --- | --- | --- | --- |
| Watching birds |  |  |  |  |
| Watching other wildlife |  |  |  |  |
| Socialising |  |  |  |  |
| Gardening |  |  |  |  |
| Growing food |  |  |  |  |
| Relaxing |  |  |  |  |
| Exercising |  |  |  |  |
| Use by children |  |  |  |  |
| Use by pets |  |  |  |  |
| Hanging washing out |  |  |  |  |
| Storage |  |  |  |  |

4. Please indicate whether you currently own or have previously owned any of the following pets

*Please select one option from each row.*

|  | Currently own | Have previously owned | Never owned |
| --- | --- | --- | --- |
| Dog(s) |  |  |  |
| Cat(s) with access to the outdoors |  |  |  |
| Other indoor pet(s) |  |  |  |
| Caged pet(s) outdoors (e.g. rabbit, chickens) |  |  |  |
| Temporary animal(s) outdoors (e.g. wild hedgehogs that are being overwintered or rehabilitated in association with a hedgehog rescue) |  |  |  |

5. On average, how often have you seen – or have seen / heard signs of – the following animals in your garden over the past year?

*Please select one option from each row.*

|  | Daily | A few times a week | A few times a month | Less | Never |
| --- | --- | --- | --- | --- | --- |
| Badgers |  |  |  |  |  |
| Foxes |  |  |  |  |  |
| Hedgehogs |  |  |  |  |  |
| Rodents (e.g. rats and mice) |  |  |  |  |  |

6. Please indicate whether you think each of the following species has increased or decreased in abundance over the course of the last 5 years in your neighbourhood.

*If you believe the species in question has always been absent, please select "stayed the same".*

*If you have not lived in your house for five years or more, please answer “not lived here long enough”.*

|  | Decreased | Stayed the same | Increased | Not lived here long enough | Unsure |
| --- | --- | --- | --- | --- | --- |
| Badgers |  |  |  |  |  |
| Foxes |  |  |  |  |  |
| Hedgehogs |  |  |  |  |  |
| Rodents |  |  |  |  |  |

7. Do you currently have any of the following wildlife-friendly features in your garden?

*Please select one option from each row.*

|  | Yes | No | No – but would consider having |
| --- | --- | --- | --- |
| Flowering lawn |  |  |  |
| Wildflowers |  |  |  |
| Wild patch |  |  |  |
| Hedgerow |  |  |  |
| Log pile |  |  |  |
| Pond |  |  |  |
| Bird box |  |  |  |
| Bat box |  |  |  |
| Hedgehog house |  |  |  |
| Insect hotel |  |  |  |
| Compost heap |  |  |  |
| Drinking water for animals |  |  |  |

8. How often do you leave food out for the following animals in your garden?

*Please select one option from each row.*

|  | Daily | A few times a week | A few times a month | Less | Never |
| --- | --- | --- | --- | --- | --- |
| Badgers |  |  |  |  |  |
| Foxes |  |  |  |  |  |
| Birds (from a feeder) |  |  |  |  |  |
| Birds (on the ground) |  |  |  |  |  |
| Hedgehogs |  |  |  |  |  |

9. What type of boundaries surround your back garden? (Please tick all that apply)

- Wooden fence
- Concrete or brick wall
- Wire fence
- Hedge
- Other (please specify)

10. Of those, which is the most common type of boundary around your back garden? (Please tick one) NB. Your answer should match one of those selected above.

- Wooden fence
- Concrete or brick wall
- Wire fence
- Hedge
- Other (please specify)

*Q11 for those who most commonly have wooden fences:*

11. We would like to know what sort of fence you have based upon its structure at ground level.

*Please tick all that apply.*

- Slats that go right to the ground
- Slats that sit on a wooden gravel board
- Slats that sit on a concrete gravel board
- Other (please specify)

**Part 2 – questions about hedgehog highways**

We would like to know how easy it is for hedgehogs to move into and out of your garden. We are particularly interested in whether they can enter or leave your garden through (a) **naturally occurring holes** and (b) **Hedgehog Highways**.

**Naturally occurring holes** refers to "holes through or under you garden boundaries which were not made by humans". These could have arisen in a number of different ways including, for example, as a consequence of an animal digging under your fence or pushing through a hedge, or general wear and tear of your fencing.

**Hedgehog Highways** are defined as "any hole through or under you garden boundaries which was DELIBERATELY created for the purposes of helping hedgehogs get into or out of your garden". These hedgehog highways may have been created by you personally, or your neighbour.

12. Could a hedgehog access your back garden from the front garden?

- Yes, ONLY through a naturally occurring hole (e.g. under a gate)
- Yes, ONLY through a Hedgehog Highway
- Yes, through both naturally occurring holes *and* Hedgehog Highways
- Yes, my back garden is openly connected to the front (e.g. there is no gate)
- No
- N/A (e.g. I do not have a front garden)

13. How many gardens border your own back garden? *This includes gardens on either side of your garden and/or at the back of your garden. Please remember this number, as it is important for the next question.*

14. Of those, how many neighbouring gardens could a hedgehog hypothetically access from your own? *Please enter your answers as numbers. If the answer is "none", please enter "0" in each box. NB. The total number reported below should not exceed your answer to the question above.*

|  | Number (type in) |
| --- | --- |
| Through ONLY a naturally occurring hole(s): |  |
| Through ONLY a Hedgehog Highway(s): |  |
| Through BOTH a naturally occurring hole(s) and Hedgehog Highway(s): |  |

15. To the best of your knowledge, how many Hedgehog Highways have been made by someone else (e.g. neighbour) that directly lead into your own back garden?

*This excludes Hedgehog Highways made by yourself. Please enter your answer as a number. If the answer is "none", enter "0".*

|  | Number (type in) |
| --- | --- |
| # Hedgehog Highways made by neighbour(s): |  |
| # Hedgehog Highways made by previous resident(s): |  |
| # Hedgehog Highways made by developer/builder(s): |  |

16. Have you or anyone currently living at your address personally made any Hedgehog Highways in your back garden?

- Yes
- No

*TWO PATHWAYS DEPENDING ON ANSWER TO Q16*

*Pathway 1 – respondents who have made highways*

17. How many Hedgehog Highways have you or anyone living at your address made in your back garden?

*Please enter your answer as a whole number. If the answer is "none", enter "0".*

18. In what year did you make your first Hedgehog Highway at your current address?

19. Did you make your first Hedgehog Highway *before* or *after* becoming a Hedgehog Champion?

- Before signing up to become a Hedgehog Champion
- After I signed up to become a Hedgehog Champion
- I am not a Hedgehog Champion

20. What motivated you to create a Hedgehog Highway? Please tick all that apply.

- I saw a hedgehog in my garden
- I had noticed a lack of hedgehogs in my garden
- The decline of hedgehog numbers in the UK
- The decline of wildlife in general in the UK
- Hearing about the Hedgehog Street campaign
- Hearing about hedgehogs on TV / in other media
- A neighbour or friend recommended constructing a Hedgehog Highway
- It was a good activity to carry out with children
- A desire to make my garden more wildlife-friendly
- Other – please specify

21. Did you create your Hedgehog Highway(s) with or without knowing that hedgehogs were visiting the *local area*?

- I created my Hedgehog Highway(s) knowing that hedgehogs were already visiting the **local area**
- I created my Hedgehog Highway(s) *without* knowing whether hedgehogs were visiting the **local area**

22. And in particular, did you create your Hedgehog Highway(s) with or without knowing that hedgehogs were visiting *your garden*?

- I created my Hedgehog Highway(s) knowing that hedgehogs were already visiting **my garden**
- I created my Hedgehog Highway(s) *without* knowing whether hedgehogs were visiting **my garden**

23. Have you observed a hedgehog(s) using your Hedgehog Highways(s)?

- Yes, I (and/or people living at my address/neighbours) have directly observed a hedgehog walking through my Hedgehog Highway(s)
- Yes, I saw a hedgehog using the Hedgehog Highway on a trail camera
- Yes - I have not seen hedgehogs using the highway directly, but I have seen other evidence that they have (e.g. footprints)
- No

24. Since making your Hedgehog Highway(s), in your opinion, how has hedgehog activity changed in your garden and the local area on the whole?

|  | Decreased | Stayed the same | Increased | Unsure |
| --- | --- | --- | --- | --- |
| Hedgehog activity in **my** **garden** has… |  |  |  |  |
| Hedgehog activity in **the local area** has… |  |  |  |  |

We would like to know whether you have been able to encourage any other households in your neighbourhood to create hedgehog highways. We are particularly interested in whether you have been successful in influencing people in (a) **your own block of houses** and / or (b) **elsewhere**.

**Your own block of houses** consists of the contiguous set of houses on your street - and any connecting streets - where the back gardens are linked. From a hedgehog's perspective, if all the gardens in your block of houses had Hedgehog Highways, then it would be able to access every garden without ever having to cross a road! The images below show several examples of blocks of houses with connecting gardens, highlighted in green.


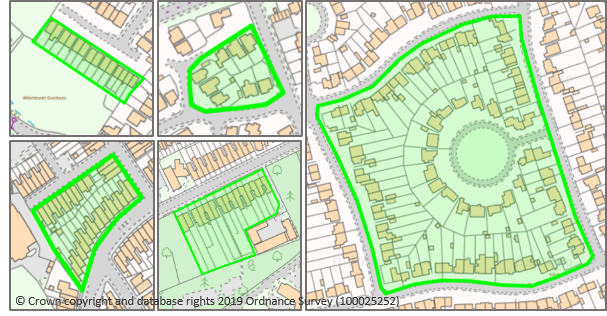


25. Have you been able to encourage **any other households in your block** into making their own Hedgehog Highways? If so, how many?

- Yes, 1 household
- Yes, 2 households
- Yes, 3 households
- Yes, 4 households
- Yes, 5 or more households
- Yes I tried, but they were not interested
- Yes I tried, but I do not know whether my neighbours followed through with it
- No, my dwelling does not form part of a block of houses
- No, and I am unlikely to try to encourage others in the future
- No, but I am likely to try to encourage others in the future

26. Have you been able to encourage any other households ***further away* from your immediate area** into making their own Hedgehog Highways? If so, how many?

*(For example, this might include friends or family that do not live on your street nor ‘block’)*

- Yes, 1 household
- Yes, 2 households
- Yes, 3 households
- Yes, 4 households
- Yes, 5 or more households
- Yes I tried, but they were not interested
- Yes I tried, but I do not know whether they followed through with it
- No, and I am unlikely to try to encourage others in the future
- No, but I am likely to try to encourage others in the future

*Pathway 2 – people who have not made highways*

We would like to know whether you have been able to encourage any other households in your neighbourhood to create hedgehog highways. We are particularly interested in whether you have been successful in influencing people in (a) **your own block of houses** and / or (b) **elsewhere**.

**Your own block of houses** consists of the contiguous set of houses on your street - and any connecting streets - where the back gardens are linked. From a hedgehog's perspective, if all the gardens in your block of houses had Hedgehog Highways, then it would be able to access every garden without ever having to cross a road! The images below show several examples of blocks of houses with connecting gardens, highlighted in green.


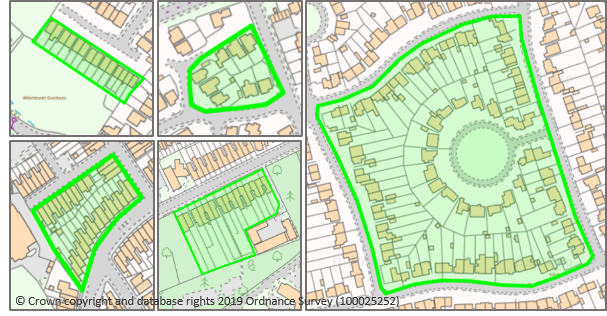


27. Although you have not made a Hedgehog Highway yourself, have you been able to encourage any other households in your block into making their own Hedgehog Highways? If so, how many?

- Yes, 1 household
- Yes, 2 households
- Yes, 3 households
- Yes, 4 households
- Yes, 5 or more households
- Yes I tried, but they were not interested
- Yes I tried, but I do not know whether my neighbours followed through with it
- No, my dwelling does not form part of a block of houses
- No, and I am unlikely to try to encourage others in the future
- No, but I am likely to try to encourage others in the future

28. Have you been able to encourage any other households **further away from your immediate area** into making their own Hedgehog Highways? If so, how many?

*(For example, this might include friends or family that do not live on your street nor ‘block’)*

- Yes, 1 household
- Yes, 2 households
- Yes, 3 households
- Yes, 4 households
- Yes, 5 or more households
- Yes I tried, but they were not interested
- Yes I tried, but I do not know whether my neighbours followed through with it
- No, and I am unlikely to try to encourage others in the future
- No, but I am likely to try to encourage others in the future

29. What are the main reasons you have not made a Hedgehog Highway yourself?

*Please tick all that apply.*

- There are no hedgehogs where I live
- My garden is already accessible
- I am not interested
- I rent my house so am not allowed
- It might encourage rats (despite their ability to climb)
- Small pets could escape
- My neighbour owns the fence
- I don’t think my neighbour would like it
- I don’t want to damage the boundary structure
- It would be unsightly
- I don’t have enough time
- I don’t have the right tools
- Other (please specify)

30. Do you plan on making a Hedgehog Highway in the future?

- Yes, likely over this winter
- Yes, likely over next spring
- Yes, likely over next summer
- Yes, but further into the future
- No
- Undecided

**Part 3 – questions about you**

The following questions relate to information about **you.** Your answers to these questions will help us find out what type of people are becoming Hedgehog Champions and whether our Champions are representative of the wider community. They will also help us to understand what might motivate different people into helping hedgehogs and ultimately help us recruit more Champions to help hedgehogs more widely.

31. Are you a member or involved with any environmental groups or wildlife charities (e.g. RSPB, National Trust, WWF) other than Hedgehog Street?

- No

- Yes (please specify)

32. Please enter your postcode below:

33. Approximately how long have you lived at your current address? Please round your answer to the nearest year.

34. How would you classify the position of your current home?

- Isolated
- In a small hamlet
- In a village
- In a town (suburban areas/fringes)
- In a town (urban centre)
- In a city (suburban areas/fringes)
- In a city (urban centre)

35. What type of house do you live in?

- Detached
- Semi-detached
- Mid-terrace
- End-of-terrace
- Flat
- Other (please specify)

36. Please indicate whether your property has the following:

*Please tick all that apply.*

- Private front garden
- Private back garden
- Communal garden
- No garden

37. Approximately how old were you when you signed up to become a Hedgehog Champion?

- 18-24
- 25-30
- 31-40
- 41-50
- 51-60
- 61+
- Prefer not to say/ I am not a Hedgehog Champion

38. How many people live in your house?

*Please enter your answer as a number. If the answer is “none”, please enter “0”.*

- Number of adults (18+): ____
- Number of children (<18): ____
- Number of temporary residents (e.g. Students home for the holidays): ____

39. What is your employment status?

*This information will help us to look into how employment and other social factors might affect people's involvement with hedgehog conservation, if at all. This sort of insight will be useful in informing future hedgehog conservation strategies.*

- Work full-time
- Work part-time
- Unemployed
- Homemaker/stay at home parent
- Student
- Retired
- Prefer not to say

*Q40 (For those who answered employed to Q39):*

40. Please select the option that best represents your occupation. For this question we are using the same divisions as outlined by the Office of National Statistics (ONS).

*This information will help us to look into how employment and other social factors might affect people's involvement with hedgehog conservation, if at all. This sort of insight will be useful in informing future hedgehog conservation strategies.*

- Managers, directors and senior officials
- Professional occupation
- Associate professional and technical occupations
- Administrative and secretarial occupations
- Skilled trades occupation
- Caring, leisure and other service occupation
- Sales and customer service occupations
- Process, plant and machine operatives
- Elementary occupations
- Prefer not to say

*BACK TO ALL RESPONDENTS*

41. What have you enjoyed **most** about being a Hedgehog Champion?

*Please select one answer.*

- Knowing I’m helping hedgehogs
- Getting involved in my local community
- Learning about local wildlife
- Meeting new people
- Learning new skills
- Sharing ideas with other Champions via the forum/gallery
- Other (please elaborate)
- I am not a Hedgehog Champion

42. Do you have any suggestions on what might encourage people to create Hedgehog Highways?

43. Do you have a photo of a Hedgehog Highway to share with us? Please upload it here!

*By uploading a photo here, you are agreeing to making your photos viewable to the public. This will include you agreeing to Hedgehog Street using your imagery for promotional purposes. If you wish for us to remove these images at any time, please email hedgehogs@ptes.org.*

44. Please enter additional comments here:

**You have reached the end of the survey. If you'd like to tell us about any other Hedgehog Highways belonging to previous addresses, please fill in another survey form with your answers relating to your previous address.**

Custom thank you:

Thank you for completing our survey! Your answers will be used to help us improve our understanding of hedgehogs and how we can help them with Hedgehog Highways and other conservation techniques.

Keep an eye on our e-news for an update on the results.
